# Modulation of TNF-α mRNA stability by human antigen R and miR181s in sepsis-induced immunoparalysis

**DOI:** 10.15252/emmm.201404797

**Published:** 2014-12-22

**Authors:** Cao Dan, Bian Jinjun, Hua Zi-Chun, Ma Lin, Chen Wei, Zhang Xu, Zhou Ri, Cheng Shun, Sun Wen-Zhu, Jiao Qing-Cai, Yin Wu

**Affiliations:** 1 The State Key Lab of Pharmaceutical Biotechnology, College of life Sciences, Nanjing UniversityNanjing, China; 2 Department of Anesthesiology and Intensive Care Unit, Changhai Hospital, Affiliated Hospital of the Second Military Medical UniversityShanghai, China; 3 The State Key Lab of Natural Medicine, China Pharmaceutical UniversityNanjing, China; 4 Jiangsu Key Lab of Pediatric Respiratory Disease, Nanjing University of Chinese MedicineNanjing, China

**Keywords:** human antigen R, immunoparalysis, microRNA181, ouabain, tumor necrosis factor α

## Abstract

Immunoparalysis is an important pathological mechanism in sepsis. However, an effective small molecule therapy is lacking. Here, we show that ouabain, a Na^+^,K^+^-ATPase ligand, can reverse immunoparalysis *in vitro*, *in vivo*, and in clinical samples. Notably, the effect of ouabain was critically dependent on TNF-α expression. However, ouabain had opposing effects on the stability of TNF-α mRNA: Ouabain triggered miR-181 transcription, which promoted TNF-α mRNA degradation and induced immunoparalysis, and ouabain triggered the nuclear export of human antigen R (HuR), which stabilized TNF-α mRNA and suppressed immuno-paralysis. Interestingly, because the miR-181 binding site is located within the HuR binding site in the 3′-untranslated region of TNF-α, in ouabain-treated cells, HuR competed with miR-181 for binding to TNF-α mRNA and recruited TNF-α mRNA to stress granules, thereby stabilizing TNF-α mRNA and reversing immunoparalysis. Ouabain also induced GM-CSF and interferon-γ expression in a HuR-dependent manner. Hence, the fine-tuning of TNF-α mRNA stability by HuR and miR181 plays a crucial role in immunoparalysis, and Na^+^,K^+^-ATPase ligands are promising agents for immunoparalysis therapy.

## Introduction

Severe sepsis leads to significant morbidity and mortality in critically ill patients and is thus an important public health issue (Rittirsch *et al*, 2008). Sepsis is characterized as an uncontrolled systematic inflammatory response. However, clinical therapies targeting inflammatory cytokines such as tumor necrosis factor (TNF)-α and interleukin (IL)-1β antagonists have shown no benefit or, in some cases, have worsened survival, suggesting a complex pathological mechanism for sepsis (Echtenacher *et al*, 2003; Moore *et al*, 2003).

A compensatory anti-inflammatory response to counter-regulate the immune response in sepsis can result in a phase of immuno-paralysis (Muenzer *et al*, 2010), which is characterized by decreased human leukocyte antigen-DR (HLA-DR) expression, impaired TNF-α production (Volk *et al*, 1996, 2000), immune effector cell apoptosis, a shift from a T_H_1 to a T_H_2 immune phenotype, and upregulation of T_reg_ cells (Oberholzer *et al*, 2001; Hotchkiss & Karl, 2003). Immunoparalysis has been convincingly established as a major pathogenic mechanism in sepsis (Hotchkiss *et al*, 2013).

Owing to the compromised immunological defense in sepsis, immunostimulants have proved to be useful. For instance, treatment with recombinant granulocyte–macrophage colony-stimulating factor (GM-CSF) has been shown to restore HLA-DR expression, leading to shorter hospital stays for patients with severe sepsis (Meisel *et al*, 2009). Similarly, treatment of immune-suppressed sepsis patients with interferon γ (IFN-γ) significantly improves monocyte immune functions and HLA-DR expression (Docke *et al*, 1997). With the exception of these protein drugs, few small molecules for the treatment of sepsis-induced immunoparalysis have been described.

Na^+^,K^+^-ATPase is involved in a number of inflammatory disorders (Eisenhut, 2006; Vadasz *et al*, 2007). Elevated levels of endogenous Na^+^,K^+^-ATPase ligands, including digoxin and ouabain, have been observed in inflammation-related diseases, including sepsis (Berendes *et al*, 2003). Na^+^,K^+^-ATPase ligands, also called cardiac glycosides, are a diverse family of naturally derived compounds that bind to and inhibit Na^+^,K^+^-ATPase. Members of this family, such as digoxin, have a long history of clinical use for the treatment of heart failure and atrial arrhythmia. Na^+^,K^+^-ATPase ligands have been implicated in the regulation of many important physiological and pathological states (Prassas & Diamandis, 2008).

Since its discovery, endogenous ouabain has been shown to participate principally in the remolding of the cardiovascular system (Nicholls *et al*, 2009). However, ouabain can also act as an immunoregulator (Matsumori *et al*, 1997; Padilha *et al*, 2008; Bagrov *et al*, 2009; Rodrigues-Mascarenhas *et al*, 2009) and as a mammalian endocrine hormone (Nicholls *et al*, 1995). We previously reported that Na^+^,K^+^-ATPase ligands can regulate cytokine expression at the post-transcriptional level (Feng *et al*, 2011), a finding that opens new avenues for understanding the pathophysiological role of Na^+^,K^+^-ATPase ligands. In this study, we show that ouabain reverses sepsis-induced immunoparalysis by reprogramming the expression of immunostimulatory cytokines at the post-transcriptional level.

## Results

### Reversal of immunoparalysis by the Na^+^,K^+^-ATPase ligand ouabain

**Figure 1:**
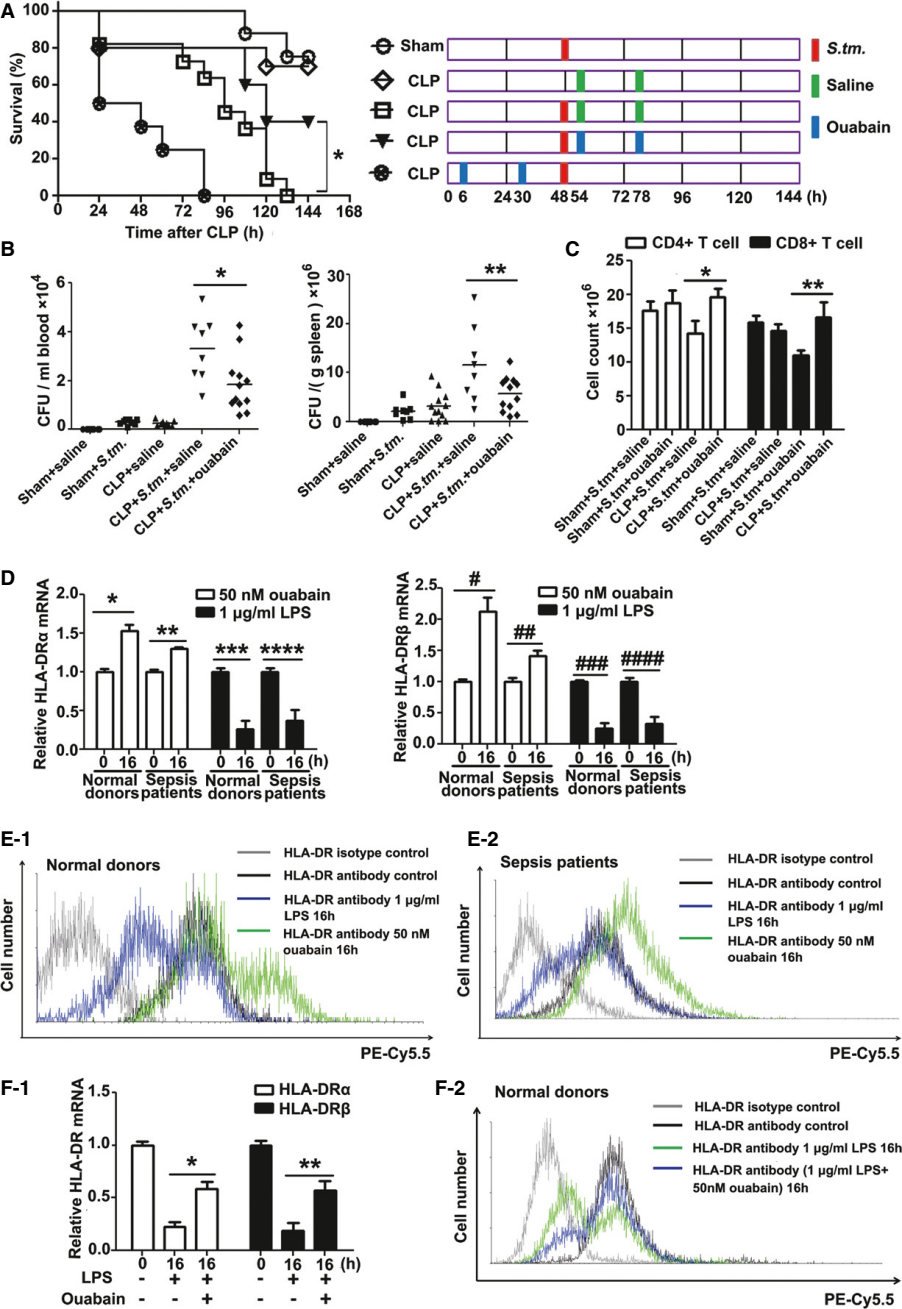
Ouabain reverses immunoparalysis in sepsis Effect of ouabain on the mortality of animals in “two-hit” sepsis model. Groups of mice were subjected to moderate CLP (punctured once about 20% mortality within 48 h), followed 2 days later by infection with *S.tm*. (2 × 10^3^ CFU, i.p.). CLP alone without *S.tm*. infection served as a control. After *S.tm*. infection, mice received saline or ouabain (0.1 mg/kg, i.p.) at 54 and 78 h, and the drug scheme was shown on the right in which a red box indicates infection with *S.tm*., green box indicates injection with saline, and blue box indicates injection with ouabain. Sham-operated mice infected with *S.tm*. (sham + *S.tm*.) were used as a control. Mice were also injected with ouabain (0.1 mg/kg, i.p.) at 6 h and 24 h after CLP, but before *S.tm*. infection. Survival after CLP and *S.tm*. infection of ouabain-treated versus untreated mice was compared (*P <* *0.05) (log-rank test). Data shown are from one experiment (*n *=* *8–12 mice per group), representative of a total of three independent experiments.

Impaired host immunity by cecal ligation and puncture (CLP) has been described in a previous study (Echtenacher *et al*, 2003) and is demonstrated here (Supplementary Fig S1A and B). We established a clinically relevant “two-hit” model of sepsis, consisting of CLP followed by the induction of *Salmonella enterica* Serovar *typhimurium* (*S.tm*.) infection. In this model, mice infected with *S.tm*. 48 h post-CLP displayed a significant increase in mortality when compared with mice subjected to *S.tm*. infection or CLP alone (Fig1A). Ouabain, however, significantly improved survival when administered at a low dose of 0.1 mg/kg at 54 and 78 h post-CLP (*P *<* *0.05). Surprisingly, when ouabain was administered before *S.tm*. infection, at 6 and 30 h after CLP, increased mortality was observed.

Bacterial loads in the blood (left panel) and spleen (right panel) of mice were measured at 48 h after infection with *S.tm.,* as described in the method section. Data shown are from one experiment (*n *=* *6–12 mice per group), representative of a total of three independent experiments. *P =* *0.001, **P =* *0.0038 (one-way ANOVA).

Ouabain suppressed the decreases in splenic CD4^+^ and CD8^+^ T cells in “two-hit” sepsis model. Splenic CD4^+^ and CD8^+^ T cells were calculated at 48 h after infection with *S.tm*., as described in the Materials and Methods section. Data shown are the mean ± SD from two independent experiments (*n *=* *8–12 mice total per condition). *P =* *0.0028, **P =* *0.0014 (one-way ANOVA).

Effects of ouabain and LPS on HLA-DR α (left panel) and β (right panel) mRNA expression in human monocytes. Data shown are the mean ± SD from two independent experiments (*n *=* *10 samples total per condition). *P =* *0.0004, **P =* *0.0002, ***P =* *0.0004, ****P =* *0.0017, ^#^*P *=* *0.0009, ^##^*P *=* *0.0035, ^###^*P *=* *0.0002, ^####^*P *=* *0.0009 (Student's *t*-test).

Flow cytometric analysis of effects of ouabain and LPS on HLA-DR surface expression in monocytes from normal donors (E-1) and sepsis patients (E-2). The experiments were performed in triplicate.

Ouabain reversed LPS-induced suppression of HLA-DR mRNA (F-1) and protein (F-2) expressions in monocytes from normal donors. Data shown are the mean ± SD from three independent experiments. *P =* *0.0001, **P =* *0.0017 (one-way ANOVA).

**Figure 2:**
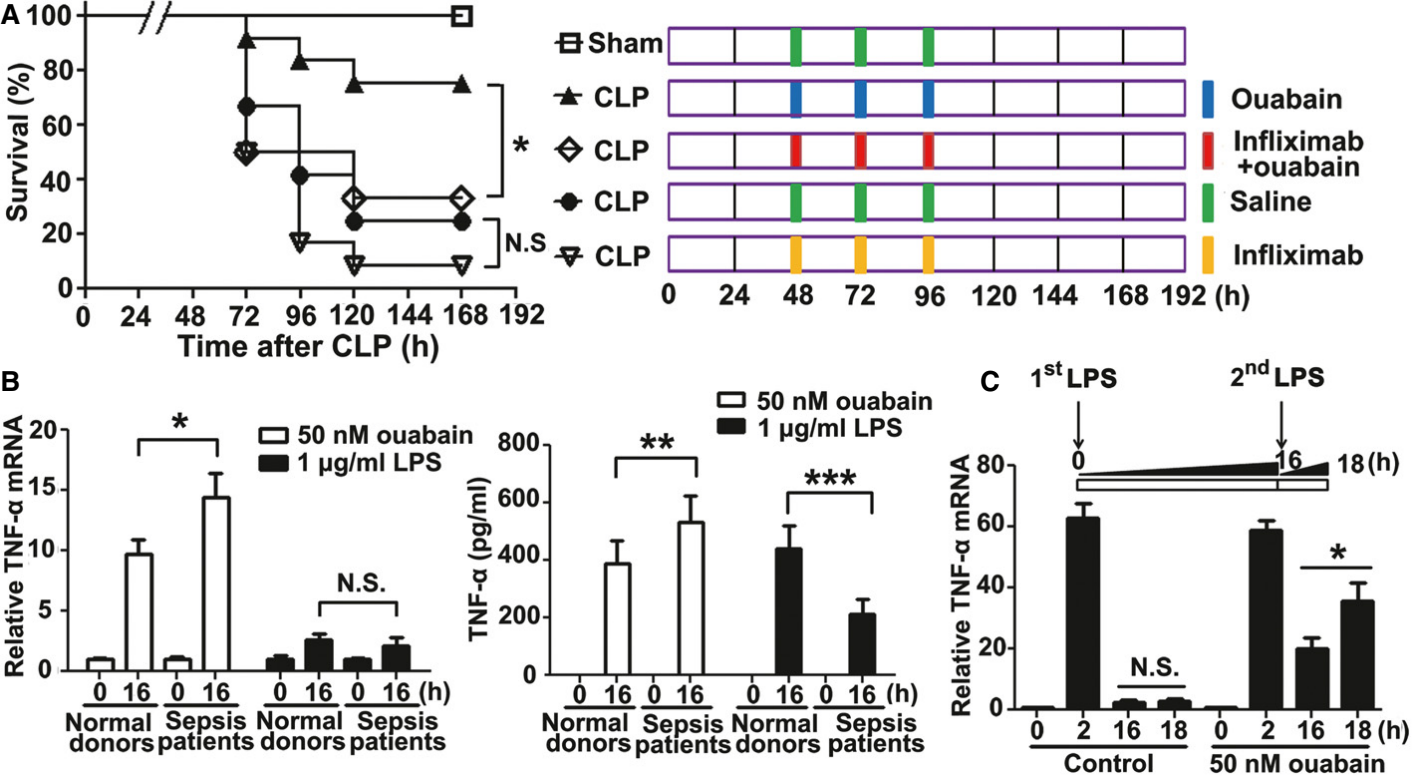
Reversal of immunoparalysis by ouabain is dependent on TNF-α Animal survival by ouabain treatment in the CLP sepsis model was blocked by infliximab (300 μg/kg, i.p.). The mortality of mice after CLP 48 h was controlled at 30–40% (punctured twice). Data shown are from one experiment (*n *=* *20 mice per group, except *n *=* *6 for sham), representative of a total of three independent experiments. *P <* *0.05 (0.0384) (log-rank test); N.S., not statistically significant.

The immunoparalysis caused by sepsis is associated with a decrease in the host's ability to clear bacteria. As shown in Fig1B, ouabain improved bacterial clearance from the blood (left panel) and spleen (right panel) of CLP mice after *S.tm*. infection. A reduction in the number of spleen CD4^+^ and CD8^+^ T cells is a hallmark of immunoparalysis (Muenzer *et al*, 2010). Ouabain treatment also restored CD4^+^ and CD8^+^ T-cell numbers in CLP mice after *S.tm*. infection (Fig1C). Decreased monocyte HLA-DR expression is a clinical indicator of immunosuppression (Volk *et al*, 1996). Ouabain increased the expression of HLA-DR mRNA (Fig1D) and the expression of HLA-DR protein on the cell surface (Fig1E-1 and Supplementary Fig S2A) in blood monocytes isolated from normal donors and sepsis patients (Fig1D and E-2 and Supplementary Fig S2B). Furthermore, the lipopolysaccharide (LPS)-induced suppression of HLA-DR mRNA (Fig1F-1) and surface protein (Fig1F-2 and Supplementary Fig S2C) expression in human blood monocytes was attenuated by ouabain. The ouabain used in this study was routinely tested for LPS contamination using the Tachypleus Amebocyte Lysate assay (sensitivity of < 1 pg/ml). The results demonstrated that the effect of ouabain was not caused by LPS contamination and was not related to Toll-like receptor 2 and 4 (Supplementary Fig S3).

### Reversal of immunoparalysis by ouabain is dependent on TNF-α

Restoration of TNF-α expression improves survival in patients with immunoparalysis (Chen *et al*, 2000; Echtenacher *et al*, 2003). In this study, when CLP animals with or without *S.tm*. infection were challenged with infliximab (a TNF-α blocker) combined with ouabain, an increase in mortality was observed (Fig2A and Supplementary Fig S4A). As shown in Supplementary Fig S4B, infliximab reversed the beneficial effect of ouabain on bacterial clearance from the blood (left panel) and spleen (right panel) in CLP mice after *S.tm*. infection. Infliximab also blocked the increase in TNF-α protein induced by ouabain (Supplementary Fig S4C). Furthermore, in response to ouabain treatment, monocytes from severe sepsis patients produced more TNF-α mRNA (Fig2B, left panel) and cytokines (Fig2B, right panel) than did monocytes from normal donors. In contrast, monocytes from sepsis patients were less susceptible to LPS treatment in producing TNF-α protein than that from normal donors. Endotoxin tolerance is an experimental analogue of clinical immunoparalysis (Biswas & Lopez-Collazo, 2009). In this study, monocytes isolated from healthy donors were primed with LPS and challenged with a second dose of LPS. As shown in Fig2C and Supplementary Fig S5, ouabain inhibited LPS-induced endotoxin tolerance.

Effect of ouabain on TNF-α mRNA (left panel) and protein (right panel) expressions in monocytes from sepsis patients and normal donors. Data shown are the mean ± SD from two independent experiments (*n *=* *10 samples total per condition). *P =* *0.0002, **P =* *0.0059, ***P =* *0.0001 (two-way ANOVA). N.S., not statistically significant.

Ouabain reversed LPS-induced endotoxin tolerance. Monocytes from five healthy volunteer blood were pre-treated with 1 μg/ml LPS only or 1 μg/ml LPS plus 50 nM ouabain for 16 h and then re-stimulated with 1 μg/ml LPS for 2 h. TNF-α mRNA levels were determined by Q-PCR. Data are expressed as mean ± SD from three independent experiments. *P =* *0.0014 (one-way ANOVA); N.S., not statistically significant.

To characterize the effects of ouabain, the effects of LPS and ouabain on TNF-α-production in human peripheral blood monocytes (Supplementary Fig S6A), THP-1 cells (Supplementary Fig S6B), and type II alveolar epithelial A549 cells (Supplementary Fig S6C) were investigated. A549 cells were used because type II alveolar epithelial cells, as a first line of defense, play critical roles in sensing microbial pathogens and initiating innate immune responses. Moreover, the lungs are particularly vulnerable to sepsis attack because of the ample blood supply. In ouabain-treated cells, TNF-α transcription was less pronounced than in LPS-treated cells, but mRNA decay was also reduced. In human monocytes (Supplementary Fig S6A), ouabain treatment led to persistent TNF-α cytokine expression over a 24-h period. NF-κB activation mainly contributes to LPS-induced TNF-α transcription. Interestingly, we found that NF-κB activation was also responsible for ouabain-induced TNF-α gene transcription (Supplementary Fig S7A–D). Therefore, LPS and ouabain might have different regulatory effects on TNF-α gene expression at the post-transcriptional level.

### The microRNA181 family negatively regulates TNF-α mRNA stability and induces immunoparalysis

**Figure 3:**
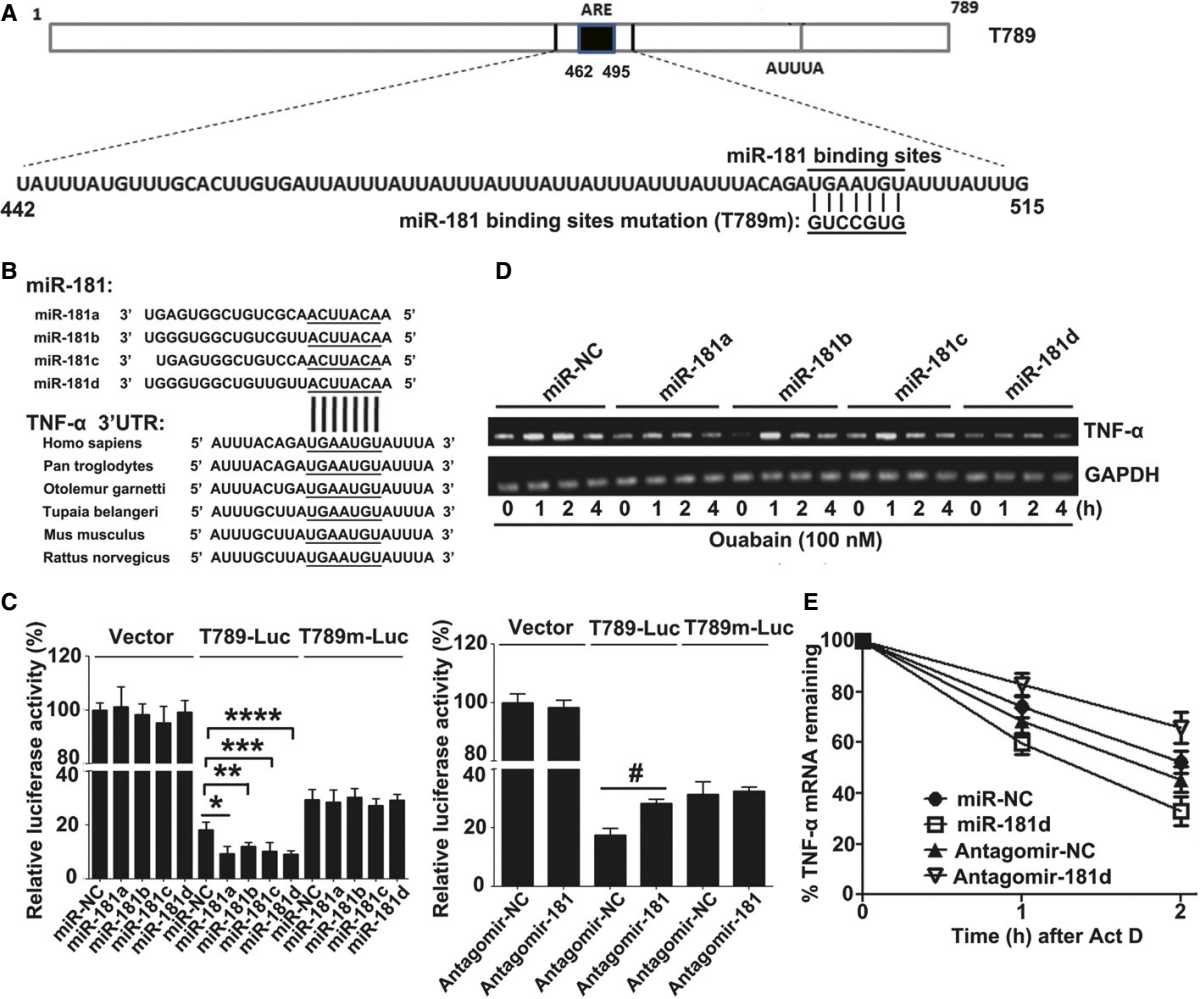
miR-181s negatively regulate TNF-α mRNA stability Human TNF-α 3′-UTR elements and potential miR-181s binding sites.

When characterizing the post-transcriptional regulation of TNF-α mRNA by ouabain, we found that the TNF-α 3′-UTR contains a potential miR-181 binding site (UGAAUGU) (Fig3A), which is highly conserved in mammals (Fig3B). The microRNA181 family consists of four members: miR-181a, miR-181b, miR-181c, and miR-181d. To verify database predictions, we showed that “mimics” of miR-181a, miR-181b, miR-181c, and miR-181d inhibited the luciferase activity of a TNF-α 3′-UTR reporter (T789), but had no effect when the miR-181 binding site was mutated (T789 m) (Fig3C, left panel). In contrast, antagomir-181s for miR-181a/b/c/d enhanced the luciferase activity of the T789 reporter, but had no effect on the T789m reporter (Fig3C, right panel).

Conserved miR-181s binding sites in the 3′-UTR of TNF-α mRNA across species.

Luciferase activity in A549 cells transfected with constructs encoding vector T789-Luc or T789 m-Luc of TNF-α 3′-UTR plus mimics of miR-181a, miR-181b, miR-181c, miR-181d (left panel), or antagomir-181 (right panel). Data are expressed as mean ± SD from three independent experiments. *P =* *0.0172, **P =* *0.0258, ***P =* *0.0319, ****P =* *0.0069, ^#^*P *=* *0.0022 (Student's *t*-test).

miR-181s suppressed ouabain-induced TNF-α gene transcription. A549 cells were transfected with miR-181s mimics or miR-NC at a final concentration of 50 nM. Twenty-four hours later, these cells were stimulated with ouabain (100 nM) for the indicated time. The TNF-α mRNAs were measured by RT–PCR and normalized to the expression of GAPDH mRNA in each sample. Data shown are representative of three independent experiments having similar results.

Effects of miR-181d and antagomir-181d on the half-life of TNF-α mRNA. A549 cells were transfected with 50 nM miR-181d mimics or 200 nM antagomir-181. Twenty-four hours later, these cells were stimulated with ouabain (100 nM) for 1 h, the culture medium was changed, and then, 5 μg/ml ActD was used to treat these cells for 1 or 2 h. The TNF-α mRNAs were measured by Q-PCR and normalized by GAPDH mRNA. Data are obtained from three independent experiments.

The miR-181 mimics also suppressed ouabain-induced TNF-α mRNA expression in A549 cells (Fig3D) and TNF-α protein expression in human blood monocytes (Supplementary Fig S8A), miR-181d had the strongest effect. Similar results were also obtained in LPS-treated A549 cells (Supplementary Fig S8B). miR-181d greatly shortened the half-life of TNF-α mRNA, while antagomir-181d extended the half-life (Fig3E). Notably, miR-181d failed to alter the general ribosome profile and TNF-α mRNA distribution in polysomes (Supplementary Fig S9A–D), indicating that members of the miR-181 family specifically regulate TNF-α mRNA stability.

**Figure 4:**
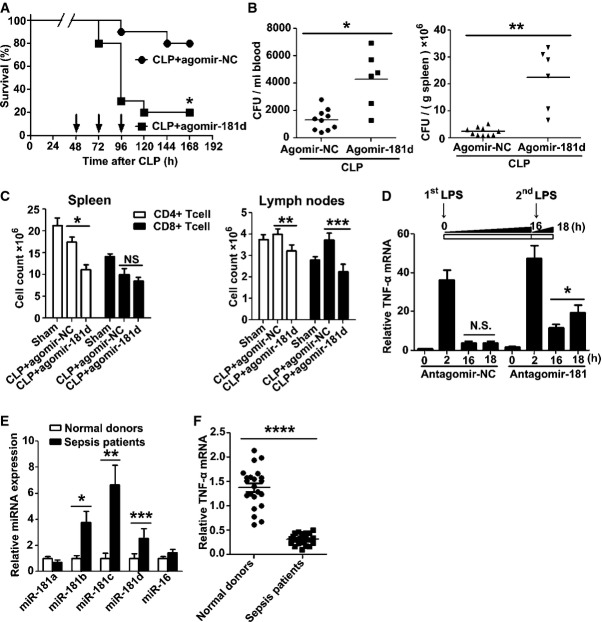
miR-181s can induce immunoparalysis Groups of mice were subjected to CLP, followed 2 days later by injection with agomir-181d or agomir-NC via tail vein at the dose of 10 nmol per mouse once a day for three consecutive days. The mortality of mice after CLP 48 h was controlled at 20% (punctured once). Data shown are from one experiment (*n *=* *20 mice per group), representative of a total of three independent experiments. *P =* *0.0042 (log-rank test).

To examine whether miR-181s could induce immunoparalysis by promoting TNF-α mRNA degradation, cholesterol-modified agomir-181d was injected into mice after CLP with or without *S.tm*. infection. Increased mortality (Fig4A and Supplementary Fig S10A) and enhanced bacterial burden in the blood (Fig4B and Supplementary Fig S10B, left panel) and spleen (Fig4B and Supplementary Fig S10B, right panel) of CLP mice were observed. Agomir-181d administration also led to a further reduction in CD4^+^ and/or CD8^+^ T cells in the spleen (Fig4C, left panel) and lymph nodes (Fig4C, right panel) of CLP mice. Moreover, administration of agomir-181d reduced the serum TNF-α levels in CLP mice with *S.tm*. infection (Supplementary Fig S10C). In contrast, cholesterol-modified antagomir-181 reduced mortality (Supplementary Fig S11A) and attenuated bacterial burden in the blood (Supplementary Fig S11B, left panel) and spleen (Supplementary Fig S11B, right panel) of CLP mice with *S.tm*. infection. Accordingly, antagomir-181 increased serum TNF-α levels in CLP mice with *S.tm*. infection (Supplementary Fig S11C). In addition, in the LPS-induced tolerance experiment, antagomir-181 treatment restored responsiveness to the LPS challenge (*P *<* *0.01) (Fig4D and Supplementary Fig S11D).

Bacterial loads in the blood (left panel) and spleen (right panel) of CLP mice after injection with agomir-181d or agomir-NC via tail vein for 2 days. Data shown are from one experiment (*n *=* *6–10 mice per group), representative of a total of three independent experiments. *P =* *0.001, **P =* *0.00003 (Student's *t*-test).

After injection with agomir-181d or agomir-NC via tail vein for 2 days, the count of CD^4+^ or CD^8+^ T cells in the spleen (left panel) and lymph nodes (right panel) of CLP mice was analyzed by flow cytometry, as described in the method section. Sham-operated mice group was used as a control. Data are expressed as mean ± SD, *n *=* *6–10 mice per group and are representative of three experiments. *P =* *0.0001, **P =* *0.0012, ***P =* *0.00004 (one-way ANOVA). N.S., not statistically significant.

Antagomir-181 reversed LPS-induced endotoxin tolerance. Monocytes from 5 healthy volunteers were isolated, pre-treated with 800 pmol antagomir-181 or matched antagomir control for 24 h, further treated with 1 μg/ml LPS for 16 h, and then re-stimulated with 1 μg/ml LPS for 2 h. TNF-α mRNA levels were determined by Q-PCR. Data are expressed as mean ± SD from two independent experiments. *P =* *0.0143 (one-way ANOVA); N.S., not statistically significant.

Inverse correlation between the expressions of miR-181s (E) and TNF-α mRNA (F) in monocytes from sepsis patients. Monocytes of 25 patients with severe sepsis and 22 healthy blood donors were isolated by adherence to plastic substrates. The culture plates were incubated for 2 h in a humidified 37°C, 5% CO_2_ incubator. After incubation, the media containing non-adherent cells were removed by aspiration. Total RNA of the adherent cells was extracted using RNeasy kit (Invitrogen, USA). miR-181s and TNF-α mRNA levels were determined by Q-PCR. Data are expressed as mean ± SD. *P =* *0.0054, **P =* *0.0034, ***P =* *0.0309, ****P =* *0.00000 (Student's *t*-test).

MicroRNAs can serve as potential biomarkers for sepsis (Sun *et al*, 2012). Therefore, we compared the expression of the miR-181 family members in human monocytes isolated from 25 patients with severe sepsis due to infections. Q-PCR analysis showed that miR-181b, miR-181c, and miR-181d were upregulated in sepsis patients, while miR-181a and miR-16 were unchanged (Fig4E). Accordingly, the TNF-α mRNA levels in monocytes from these patients were downregulated (Fig4F).

### LPS and ouabain trigger miRNA-181c/d transcription through Egr-1

**Figure 5:**
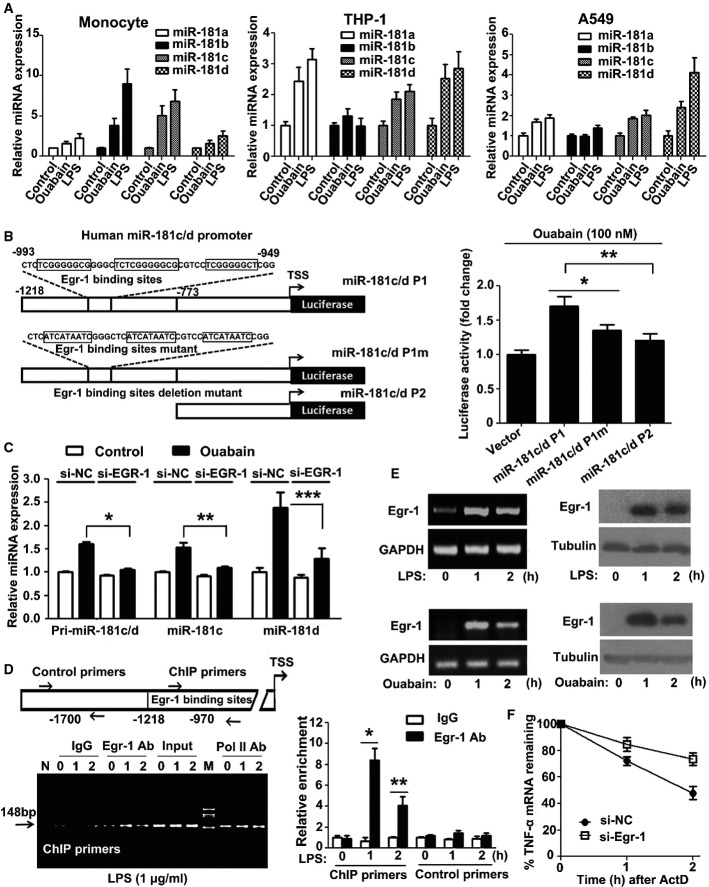
LPS and ouabain trigger miR-181c/d transcription through Egr-1 Ouabain and LPS induced miR-181s expression in human blood monocytes (left panel), THP-1 cells (middle panel), and A549 cells (right panel). Data are obtained from three independent experiments and expressed as mean ± SD.

LPS and ouabain rapidly stimulated the expression of the miR-181s in human monocytes (Fig5A, left panel), THP-1 cells (Fig5A, middle panel), and A549 cells (Fig5A, right panel). Analysis of the genetic location of the human miR-181s showed that the miR-181a-1/b-1 and miR-181a-2/b-2 clusters are on chromosomes 1 and 9, respectively, in intronic regions. However, the miR-181c/d cluster is located in an intergenic region on chromosome 19, and miR-181d is slightly downstream of miR-181c (Supplementary Fig S12A). miR-181c and miR-181d are co-transcribed (Supplementary Fig S12B and C). The transcriptional start site of miR-181c was previously determined using 5′-RACE (Hashimoto *et al*, 2010). Three potential early growth response protein 1 (Egr-1) binding sites are present between nucleotides −993 and −949 in the human miR-181c/d promoter (Fig5B, left panel). To determine the role of the Egr-1 binding sites in miR-181c/d transcription, cells were transiently transfected with human miR181c/d promoter deletion (miR-181c/d P2) or mutation (miR-181c/d P1 m) constructs. As expected, the increases in miR-181c/d promoter activity induced by ouabain (Fig5B, right panel) or LPS (Supplementary Fig S12D) were attenuated when the Egr-1 binding sites were mutated or deleted. Furthermore, when siRNA was used to knock down Egr-1 expression, the ouabain-induced increase in pri-miR-181c/d, mature miR-181c, and mature miR-181d expression was attenuated (Fig5C). Chromatin immunoprecipitation **(**ChIP) analysis confirmed that Egr-1 associated with the miR-181c/d promoter after LPS treatment (Fig5D). Both LPS (Fig5E, upper panel) and ouabain (Fig5E, below panel) induced Egr-1 mRNA and protein expression. Knockdown of Egr-1 expression accelerated TNF-α mRNA decay (Fig5F). Taken together, these results indicate that Egr-1 plays an important role in miR-181c/d transcription.

Construction of firefly luciferase reporter plasmid harboring miR-181c/d promoter and its mutants (left panel). The luciferase activity (firefly/*Renilla*) of cells transfected with miR-181c/d promoter or its mutant (right panel). A549 cells were transiently transfected with 1 μg firefly luciferase plasmids and 50 ng pRL-TK and then treated with 100 nM ouabain for an additional 8 h. The luciferase activity (firefly/*Renilla*) of each transfection group is indicated. The luciferase activity of vector was arbitrarily set at 1.0. Data represent the mean ± SD from three independent experiments. *P =* *0.0005, **P = 0.00005 (one-way ANOVA).

The expression levels of pri-miR-181c/d, miR-181c, and miR-181d after Egr-1 siRNA transfection. A549 cells were transfected with EGR1 siRNA or control siRNA. Twenty-four hours later, these cells were stimulated with 100 nM ouabain for 2 h. Q-PCR analysis was performed to measure the expression levels of pri-miR-181c/d, miR-181c, and miR-181d. Data represent the mean ± SD from three independent experiments. *P =* *0.0002, **P =* *0.0006, ***P =* *0.0113 (two-way ANOVA).

ChIP assay of the binding of Egr-1 to the miR-181c/d promoter. THP1 cells were stimulated for the indicated time with 1 μg/ml LPS and assessed with ChIP primer and the control primer for the promoter. Q-PCR result is shown (right panel). Lane M, DNA size markers (in bp). Lane N, distilled water was used as PCR template. Data represent the mean ± SD from three independent experiments. *P =* *0.0003, **P =* *0.0033 (Student's *t*-test).

LPS (upper panel) and ouabain (below panel) time-dependently stimulated Egr-1 mRNA and protein expression in THP1 cells. The experiments were performed in triplicate.

Silencing of Egr-1 suppressed TNF-α degradation. A549 cells were transfected with EGR1 siRNA or control siRNA. Twenty-four hours later, these cells were stimulated with 1 μg/ml LPS for 1 h, the culture medium was changed, and then 5 μg/ml ActD was used to treat these cells for 1 or 2 h. The TNF-α mRNAs were measured by Q-PCR. GAPDH was included as a control. Data represent the mean ± SD from three independent experiments.

### Ouabain-induced HuR export plays a pivotal role in regulating TNF-α mRNA stability

**Figure 6:**
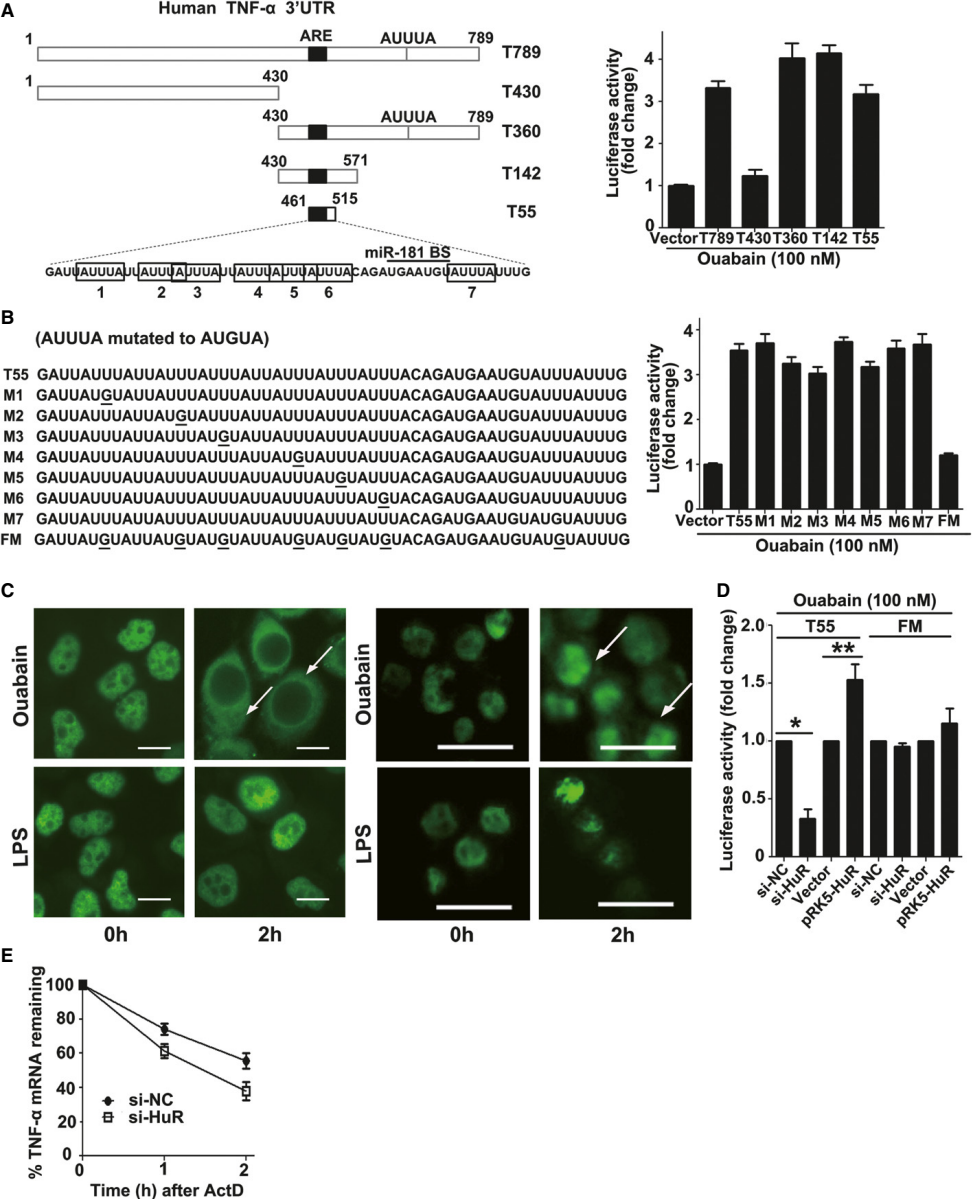
HuR increases TNF-α mRNA stability Schematic depiction of TNF-α 3′-UTR and its mutants (left panel). Identification of the minimal *cis*-element in TNF-α 3′-UTR that is responsive to ouabain treatment (A). Effect of ouabain on the luciferase activity of T55 and its mutants (B). A549 cells were transfected with T789 or its mutants. After 24 h, cells were stimulated with 100 nM ouabain for 12 h. The luciferase activity of vector control was arbitrarily set at 1.0. Data represent the mean ± SD from three independent experiments.

Although ouabain induced miR-181d expression, it still reversed immunoparalysis and delayed TNF-α mRNA decay. These conflicting results suggest that additional ouabain-dependent mechanisms counter-regulate miR181d-mediated TNF-α mRNA decay. To identify these mechanisms, full-length and truncated TNF-α 3′-UTR luciferase reporter plasmids were constructed (T789-luc, T430-luc, T360-luc, T142-luc, and T55-luc) (Fig6A, left panel). Ouabain increased TNF-α 3′-UTR luciferase activity, and T55 was the minimal *cis*-regulatory element required for ouabain's effect (Fig6A, right panel). Notably, seven “AUUUA” motif sequences exactly clustered and overlapped within the T55 region (Fig6B, left panel). The effect of ouabain on the T55 reporter varied weakly when a single “AUUUA” motif was mutated, but was almost completely suppressed when all “AUUUA” motifs were mutated (Fig6B, right panel).

Effects of ouabain and LPS on HuR translocation in A549 cells (left image) or in THP-1 cells (right image). Representative fluorescent images of three independent experiments showing similar results are shown. Scale bars: 10 μm.

Effect of HuR on the luciferase activity of T55 reporter under the condition of ouabain treatment. A549 cells were co-transfected with 50 nM HuR siRNA (or control siRNA), 1 μg T55 (or FM) firefly luciferase reporter plasmid, and 50 ng pRL-TK. Twenty-four hours later, cells were treated with ouabain at 100 nM for an additional 12 h. For HuR overexpression experiments, A549 cells were transfected with 0.5 μg vector or pRK5-HuR plasmid for 12 h and then co-transfected with 0.5 μg T55 or FM firefly luciferase reporter plasmid and 50 ng pRL-TK. After transfection, cells were treated with ouabain at 100 nM for an additional 12 h. The luciferase activity of matched control was arbitrarily set at 1.0. Data represent the mean ± SD from three independent experiments. *P = 0.0001, **P = 0.0027 (Student's *t*-test).

Effect of HuR silencing on the decay of TNF-α mRNA. A549 cells were transfected with HuR siRNA or control siRNA. Twenty-four hours later, these cells were stimulated with ouabain (100 nM) for 1 h, the culture medium was changed, and then 5 μg/ml Act D was used to treat these cells for 1 or 2 h. The TNF-α mRNAs were measured by Q-PCR. GAPDH was included as a control. Data represent the mean ± SD of three experiments.

Human antigen R (HuR) is a common RNA binding protein that regulates mRNA stability and translation efficacy (Srikantan *et al*, 2012) by binding to AU-rich elements (AREs) within the 3′-UTRs of target genes. In the present study, ouabain induced HuR translocation in A549 (Fig6C, left image) and THP-1 cells (Fig6C, right image), but no significant translocation was observed in LPS-treated cells. Furthermore, ouabain-induced T55 reporter gene expression was suppressed by HuR siRNA. In contrast, HuR overexpression enhanced luciferase activity in cells transfected with the T55 reporter, but not with T55FM (Fig6D), and HuR silencing accelerated TNF-α mRNA decay (Fig6E). These results show that HuR mediates the TNF-α mRNA stabilization induced by ouabain.

### HuR protects CLP mice from immunoparalysis

**Figure 7:**
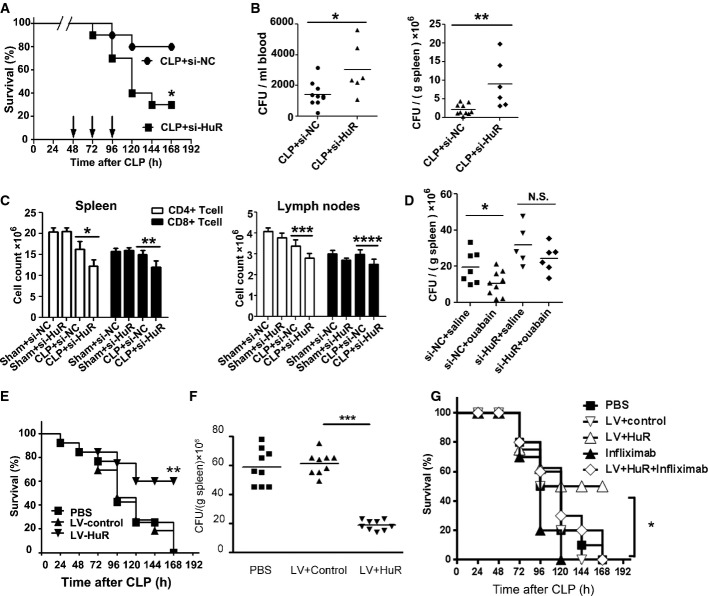
HuR protects CLP mice from immunoparalysis Groups of mice were subjected to CLP, followed 2 days later by injection with cholesterol-modified HuR siRNA or control siRNA via tail vein at the dose of 10 nmol per mouse. The mortality of mice after CLP 48 h was controlled at 20% (punctured once). Data shown are from one experiment (*n *=* *20 mice per group), representative of a total of three independent experiments. *P <* *0.05 (log-rank test).

To examine whether HuR can reverse immunoparalysis, mice were challenged with cholesterol-modified HuR siRNA after CLP with or without *S.tm*. infection. The results, shown in Fig7A and Supplementary Fig S13A, showed that HuR siRNA administration increased CLP mortality (*P *<* *0.05) and increased the bacterial burden in the blood (Fig7B and Supplementary Fig S13B, left panel) and spleen (Fig7B and Supplementary Fig S13B, right panel). HuR silencing decreased the CD4^+^ and CD8^+^ T-cell populations in the spleen (Fig7C, left panel) and lymph nodes (Fig7C, right panel) of CLP mice. Moreover, the serum TNF-α levels in CLP mice with *S.tm*. infection were reduced by treatment with HuR siRNA (Supplementary Fig S13C). Importantly, the improved bacterial clearance observed after ouabain treatment in CLP mice was attenuated by exposure to HuR siRNA (Fig7D). Highlighting the effect of HuR, lentiviral overexpression of HuR prolonged survival (Fig7E) and improved bacterial clearance in the spleen of mice after CLP and *S.tm* infection (Fig7F). Moreover, the beneficial effect of HuR overexpression on survival in mice with CLP and *S.tm* infection was attenuated by infliximab, suggesting that the effect of HuR is dependent on TNF-α (Fig7G).

Bacterial loads in the blood (left panel) and spleen (right panel) of CLP mice after injection with agomir-181d or agomir-NC via tail vein for 2 days. Data are mean ± SD, *n *=* *6–10 mice per group and are representative of three experiments. *P =* *0.0203, **P =* *0.0061 (Student's *t*-test).

After injection with HuR siRNA or control siRNA via tail vein for 2 days, the count of CD4^+^ or CD8^+^ T cells in the spleen (left panel) and lymph nodes (right panel) of CLP mice or sham-operated mice was analyzed by flow cytometry. Data are expressed as mean ± SD, *n *=* *6–10 mice per group, and are representative of three experiments. *P =* *0.0006, **P =* *0.0005, ***P =* *0.0017, ****P =* *0.002 (one-way ANOVA).

Bacterial loads in the spleen of CLP mice. At 2 days after CLP, animals were injected with HuR siRNA (10 nmol/mouse) via tail vein and ouabain (0.1 mg/kg, i.p.) once a day for three consecutive days. Data are obtained from one experiment, representative of a total of three independent experiments. *P =* *0.0388 (Student's *t*-test). N.S., not statistically significant.

Groups of mice were subjected to moderate CLP (punctured once about 10–20% mortality within 48 h), followed 2 days later by infection with *S.tm*. (2 × 10^3^ CFU, i.p.). Mice were further challenged with PBS or lentiviruses containing control or HuR expressing plasmid (10^8^ TU per mice) at 54, 78, and 102 h after CLP via tail vein. Survival of CLP mice after injection with lentiviruses containing HuR-overexpressing plasmid versus control plasmid was compared (**P <* *0.01, log-rank test). Bacterial loads in the spleen of CLP mice are shown in (F). *n *=* *9 mice per group. ***P <* *0.001, N.S., not statistically significant. Data represent the mean ± SD from two independent experiments.

Groups of mice were treated as that in (E) and (F) and further challenged with PBS, lentiviruses containing control, or HuR-expressing plasmid (10^8^ TU per mice) with or without infliximab (300 μg/kg). Survival of CLP mice after injection with lentiviruses containing HuR-overexpressing plasmid versus HuR plus infliximab was compared (*P <* *0.001, log-rank test).

### HuR antagonizes miR-181d-mediated TNF-α mRNA destabilization

**Figure 8:**
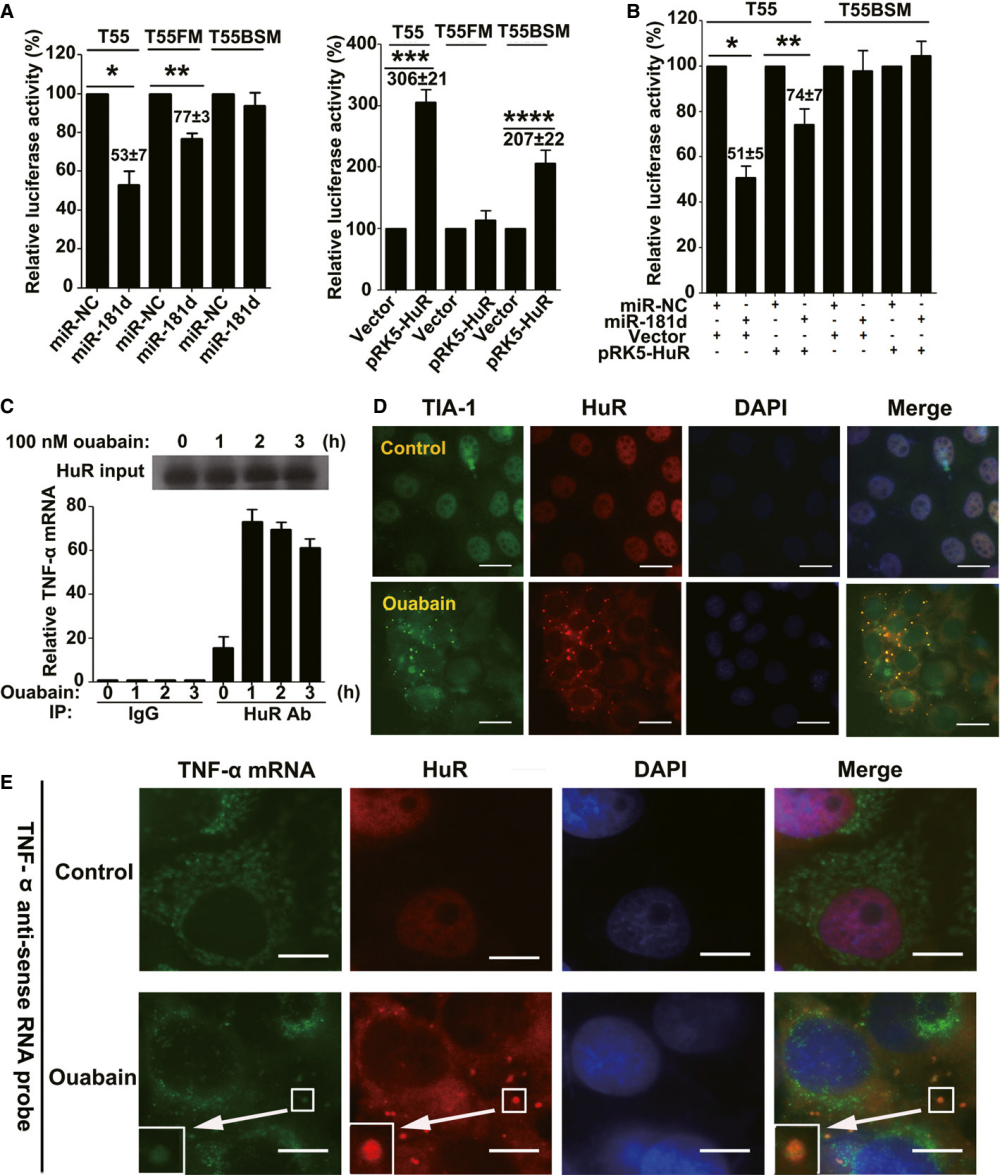
HuR competes with miR-181d on the shared target of TNF-α mRNA Effects of miR-181d (left panel) and pRK5-HuR (right panel) on the luciferase activity of cells transfected with TNF-α 3′-UTR and its mutants. A549 cells were transfected with the indicated constructs, miR-181d or pRK5-HuR. Data represent the mean ± SD from three independent experiments. *P =* *0.0004, **P =* *0.0002, ***P =* *0.00007, ****P =* *0.001 (Student's *t*-test).

Because the miR-181 binding site in the 3′-UTR of TNF-α is located within two adjacent “AUUUA” motifs (Supplementary Fig S14A), we speculated that, after ouabain treatment, HuR might counteract the destabilizing effect of miR-181d on TNF-α mRNA. As shown in Fig8A, miR-181d overexpression reduced T55 (WT) reporter luciferase activity to 53% of the activity observed in control cells; however, the effect of miR-181d on T55FM was less potent (Fig8A, left panel). Likewise, forced HuR expression increased T55 WT reporter activity up to threefold, relative to that in control cells. However, the effect of HuR on the T55 reporter with a mutated miR-181d binding site (T55BSM) was less potent (Fig8A, right panel). Thus, the AREs and the miR-181 binding site within the TNF-α 3′-UTR are *cis-*regulatory elements that are functionally dependent on each other. When we examined possible competition between the two elements, we found that HuR overexpression counteracted the effect of miR-181d on the T55 WT reporter (Fig8B). Additionally, RNA immunoprecipitation experiments demonstrated that HuR associated with cytoplasmic TNF-α mRNA, even in the absence of ouabain treatment; however, ouabain treatment increased the association (Fig8C). In a control experiment, ouabain failed to increase the association between β-actin mRNA and HuR, even though the 3′-UTR of β-actin also contains AREs (Dormoy-Raclet *et al*, 2007) (Supplementary Fig S14B).

HuR and miR-181d are mutually antagonistic for TNF-α mRNA stabilization. A549 cells were transfected with the indicated constructs, miR-181d or pRK5-HuR. Data represent the mean ± SD from three independent experiments. *P =* *0.00008, **P =* *0.0031 (Student's *t*-test).

Ouabain increased the association of cytoplasmic HuR with TNF-α mRNA. Q-PCR assay of TNF-α mRNA immunoprecipitated by HuR antibody or immunoglobulin G (IgG) from lysates of A549 cells treated with ouabain at a concentration of 100 nM for the indicated time. Top, immunoblot analysis of HuR protein input in A549 cell lysates. Data represent the mean ± SD from three independent experiments.

Immunofluorescence analysis of the SGs in A549 cells after ouabain treatment. A549 cells were challenged with ouabain (100 nM) for 6 h. SGs were visualized by counterstaining for TIA-1 (green) and HuR (red). DAPI was used for staining nuclei. Merged views are shown in the right panels. Representative fluorescent images of three independent experiments showing similar results are shown. Scale bars: 20 μm.

Ouabain recruited TNF-α mRNA to SGs. After treated or untreated with ouabain (100 nM) for 12 h, A549 cells were fixed, permeabilized, and then incubated with 3 nM of an Alexa Fluor 488-labeled antisense RNA probe to detect TNF-α mRNA. SGs were visualized by counterstaining for HuR, as indicated in red. DAPI was used for staining nuclei. Merged views are shown in the right panels. Scale bars: 10 μm. Shown are typical results from five different fields and three different experiments.

Interestingly, when cells were treated with ouabain at 100 nM for 6 h, many HuR-associated small cytoplasmic foci appeared. The foci were identified as stress granules (SGs) because of their co-localization with the SG marker T-cell-restricted intracellular antigen-1 (TIA-1) (Fig8D). To examine whether the SGs participate in TNF-α mRNA turnover, RNA-FISH was performed using specific TNF-α mRNA probes. The results, shown in Fig8E and Supplementary Fig S14C, demonstrated that a relatively large proportion of TNF-α mRNA co-localized with HuR in SGs in ouabain-treated cells, suggesting that ouabain can recruit TNF-α mRNA into SGs. To examine the biological significance of the SGs, cells were transfected with TIA-1 siRNA (Supplementary Fig S14D), which suppressed ouabain-induced SG formation (Supplementary Fig S14E). In this context, TNF-α mRNA decay in ouabain-treated cells was accelerated (Supplementary Fig S14F).

## Discussion

Using a clinically relevant “two-hit” model of sepsis, we showed that ouabain improved survival and increased bacterial clearance under conditions of immunoparalysis. Additionally, ouabain increased HLA-DRα and HLA-DRβ gene expression and restored TNF-α production in monocytes from sepsis patients. To the best of our knowledge, this is the first report to demonstrate that a small molecule drug can reverse immunoparalysis.

Ouabain is involved in regulating immunity (Bagrov *et al*, 2009) and inflammatory responses (Feng *et al*, 2011; de Vasconcelos *et al*, 2011; Kennedy *et al*, 2013). We demonstrated that the immunostimulatory effects of ouabain can be either harmful or beneficial in sepsis therapy. At the stage of immunoparalysis, ouabain treatment improved animal survival, but when administered 6 h after CLP, in a state of hyperinflammation, ouabain increased mortality (Fig1A). Therefore, immune status monitoring is necessary for the proper use of ouabain in sepsis therapy.

To date, several miRNAs, including miR-146, miR-155, and miR-125b, have been shown to induce endotoxin tolerance by affecting LPS-TLR4 signaling (Nahid *et al*, 2011). Here, we showed that members of the miR-181 family act downstream of TLR4 signaling to induce TNF-α mRNA degradation. Notably, miR-181 binding sites are frequently distributed in the 3′-UTRs of many inflammatory cytokines, including IL-1α and TNF-α; surprisingly, no miR-181 binding sites have been found in the 3′-UTRs of anti-inflammatory cytokines such as IL-10 and TGF-β. In light of this, we presume that miR-181 upregulation is an important host protective mechanism against endotoxin shock, because it can shift the immune status from hyperinflammation to endotoxin tolerance via a rapid shutdown of inflammatory cytokine expression without altering anti-inflammatory cytokine expression.

The reversal of immunoparalysis by ouabain involved HuR. HuR competed with miR-181d for binding to a shared TNF-α mRNA target sequence, leading to TNF-α mRNA stabilization. Because HuR and argonaute share many interacting mRNAs, HuR and microRNAs can function competitively and cooperatively to regulate target mRNA stability or translation efficiency (Srikantan *et al*, 2012). In ouabain-treated cells, TNF-α mRNA stability was preferentially regulated by HuR and not by miR-181 family members. In fact, only one miR-181 binding site is present in the minimal TNF-α 3′-UTR (T55); in contrast, seven “AUUUA” motifs are located in the immediate vicinity of the miR-181 binding site. Therefore, it is likely that the binding of HuR to the 3′-UTR of TNF-α triggers a conformational change in the local RNA that masks the miR-181 binding site.

HuR is involved in an important negative regulatory mechanism for immunoparalysis. The failure of LPS to trigger HuR nuclear export is understandable: Without HuR export, LPS rapidly induces endotoxin tolerance for host protection against endotoxin injury. However, although the absence of HuR translocation is likely beneficial at the initial stage of hyperinflammation, this effect is deleterious at the stage of immunoparalysis because the mRNAs of many immunostimulatory cytokines, including TNF-α, IFN-γ, and GM-CSF, contain classic “AUUUA” motifs within their 3′-UTRs, and their expression is thus tightly controlled by HuR. Ouabain supplementation during immunoparalysis can remedy the deficiency in HuR translocation, as shown by the increase in GM-CSF and IFN-γ mRNA expression induced by ouabain in human monocytes during LPS-induced endotoxin tolerance experiments (Supplementary Fig S15A and B). Likewise, ouabain preferentially upregulated GM-CSF and IFN-γ mRNA expression in monocytes isolated from sepsis patients; the effect on monocytes from healthy individuals was not as strong (Supplementary Fig S15C and D). In addition, decay of GM-CSF and IFN-γ mRNA was accelerated by HuR silencing in ouabain-treated human monocytes (Supplementary Fig S15E and F). GM-CSF and IFN-γ have been shown to reverse immunoparalysis by increasing HLA-DR expression and restoring antigen-presenting activity in monocytes (Docke *et al*, 1997; Meisel *et al*, 2009). Consistently, ouabain increased HLA-DR expression in monocytes from sepsis patients. Thus, via HuR translocation, ouabain might stabilize the mRNA of many immunostimulatory cytokines, including TNF-α, GM-CSF, and IFN-γ, thereby reprogramming cytokine expression in the immunosuppressive state and reversing immunoparalysis.

In this study, ouabain reversed not only the impairment in TNF-α production in monocytes, but also the sepsis-induced reduction in CD4^+^/CD8^+^ T cells. It appeared that the effect of ouabain on CD4^+^/CD8^+^ T cells was partially dependent on TNF-α (Supplementary Fig S16). A previous study suggested that low concentrations of ouabain can initiate Na^+^,K^+^-ATPase-SRC signaling to protect cells independent of Na^+^,K^+^-ATPase ion transporting activity (Bagrov *et al*, 2009). TNF-α has two receptors, TNFR1 and TNFR2. TNFR1 is mainly pro-apoptotic, but the binding of TNFR2 to TNF-α mediates anti-apoptotic signaling (Tartaglia *et al*, 1991). Ouabain analogs, including digoxin and oleandrin, can prevent the binding of TRADD to TNFR1, thus attenuating TNFR1-mediated signaling (Yang *et al*, 2005). These results suggest that ouabain protects CD4^+^/CD8^+^ T cells from apoptosis via TNF-α/TNFR2-mediated signaling. On the other hand, studies have suggested that GM-CSF and IFN-γ can suppress immune cells apoptosis in sepsis-induced immunosuppression (Docke *et al*, 1997; Williams *et al*, 1998; Hotchkiss *et al*, 2003; Leentjens *et al*, 2012). In this study, ouabain stabilized GM-CSF and IFN-γ mRNA. Thus, it is also possible that ouabain protects CD4^+^/CD8^+^ T cells via GM-CSF and IFN-γ.

Although TNF-α plays an important role in sepsis-induced immunoparalysis, it is not the only factor involved in immunoparalysis. In this study, we found that ouabain failed to improve survival completely. Given the complex pathology of sepsis, any single treatment cannot be fully effective. While ouabain could play a prominent role in revering immunoparalysis, it would produce a more potent effect when used in combination with other approaches.

In view of the importance of HuR in the reversal of immuno-paralysis, we investigated the underlying signaling events. Studies of HuR point mutants showed that serine 88 and serine 158 within HuR were critical residues for HuR nuclear export (Supplementary Fig S17A–H). We previously showed that p38-MK2 signaling mediated ouabain-induced HuR translocation (Feng *et al*, 2011), which raises the possibility that serine 88 and serine 158 are phosphorylated by p38 MAPK in cells treated with ouabain. If this is the case, modulation of p38 signaling could be a therapeutic approach for the treatment of sepsis-induced immunoparalysis. In fact, a previous study demonstrated that p38 activity was impaired in LPS-induced endotoxin tolerance (Kraatz *et al*, 1999; Carter *et al*, 2003; Nimah *et al*, 2005). In our opinion, the impairment of p38 MAPK activity likely contributes to the failure of HuR translocation in LPS-induced endotoxin tolerance or sepsis-induced immunoparalysis.

Cardiac glycosides have a number of pharmacological activities (Prassas & Diamandis, 2008). We propose here a novel therapeutic application of cardiac glycosides in the treatment of sepsis-related immunoparalysis. In a previous study, ouabain protected against LPS-induced lethal toxicity in mice (Matsumori *et al*, 1997), while intravenous digoxin improved sepsis-associated myocardial dysfunction in experimental and clinical studies (Worthley & Holt, 1999). Endogenous glycosides, including endogenous ouabain, are elevated in a significant proportion of critically ill patients (Berendes *et al*, 2003). Taken together, cardiac glycosides are promising agents for sepsis therapy that deserve further investigation.

## Materials and Methods

### Reagents and animals

Dulbecco's modified Eagle's medium (DMEM), RPMI 1640 medium, and Lipofectamine 2000 were from Invitrogen (CA, USA). Ouabain (purity ≥ 95%), actinomycin D (Act D), and LPS (O55:B5) were from Sigma (St. Louis, MO, USA). The protein content in LPS was < 3% based on the quality report. Ouabain was routinely tested for LPS contamination using the Tachypleus amebocyte lysate assay (sensitivity of < 1 pg/ml) (Chinese Horseshoe Crab Reagent Manufactory, Co., Ltd., Xiamen, China). Male C57BL/6 mice were obtained from the Laboratory Animal Center of Nanjing University (Nanjing, China), and 10 animals are employed in each group. If the body weight of animals did not fall within the standard, or if the animals were very sensitive to CLP treatment, they were excluded from experiments. The animals were randomly allocated into experimental groups without any labeling procedure, and blinding of the investigator was employed to minimize the effects of subjective bias during group allocation and when assessing results. The animals were housed in standard cages at 25°C, on a 12/12 light–dark cycle in a clean room, and they were supplied with food and water *ad libitum*. All animals received human care according to Chinese legal requirements, and experiments were approved by The Animal Experimental Committee of Nanjing University.

### Clinical sample collection

We enrolled patients (*n *=* *33) admitted for severe sepsis to the intensive care unit (Department of Anesthesia, Changhai Hospital, Affiliated Hospital of the Second Military Medical University) and compared them with healthy donors. All subjects enrolled in this study fulfilled the criteria defined by the 2001 International Sepsis Definitions Conference (Levy *et al*, 2003). Detailed pathological information on these patients is provided in Supplementary Table S1. The sepsis patients entered a phase of immunoparalysis, as indicated by a low level of mHLA-DR expression (< 30%). The experiment involving human subject was approved by the Medical Ethics Committee of Changhai hospital of China. The informed consent was obtained from all ICU patients involved in this project, and the experiments conformed to the principles set out in the WMA Declaration of Helsinki and the Department of Health and Human Services Belmont Report.

### Cell culture

Human THP-1 cells and type II lung epithelial cells (A549) were purchased from American Type Culture Collection (ATCC; Manassas, VA, USA). THP-1 cells were cultured in RPMI 1640 medium containing 10% fetal bovine serum (FBS), 10 mM glutamine, and 50 U/ml penicillin/streptomycin. A549 cells were cultured in DMEM supplemented with 10% FBS and 50 U/ml penicillin/streptomycin. All types of cells were maintained at 37°C in a humidified atmosphere of 95% air and 5% CO_2_.

### Human monocyte isolation and HLA-DR expression

Human peripheral blood mononuclear cells (PBMC) were isolated through Ficoll/Paque (Pharmacia, Uppsala, Sweden) density gradient centrifugation of heparinized blood obtained from healthy adult donors and severe sepsis patients. Cells were harvested from the interphase layer, washed twice in phosphate-buffered saline (PBS), and then resuspended in RPMI 1,640 medium containing 10% (v/v) FBS, 10 mM glutamine, and 50 U/ml penicillin/streptomycin. Total cell counts were obtained using an Invitrogen Countess™ Automated Cell Counter (Invitrogen, CA, USA). Cell viability, assessed using trypan blue, was > 90%. Cells were cultured in 6-well tissue culture plates (4 × 10^6^ cells/ml). Monocytes were isolated using the plastic adherence method (Zhou & Tedder, 1995). The culture plates were incubated overnight in a humidified incubator at 37°C with 5% CO_2_. After incubation, the medium containing non-adherent cells was removed by aspiration, and the plates were washed with PBS pre-warmed to 37°C to obtain adherent monocytes.

The expression of cell surface HLA-DR on monocytes was measured with flow cytometry. The monoclonal antibodies used were as follows: CD14-FITC (clone M5E2; BD Biosciences, San Jose, CA, USA) and PerCP-Cy™ 5.5 (clone G46-6; BD Biosciences). The negative controls were the mouse monoclonal antibodies IgG2α-FITC (clone G155-178) and IgG2α-PerCP-Cy™ 5.5 (clone G155-178), which were isotype-matched in accordance with the recommendations of the manufacturer. Monocytes were characterized based on CD14 expression. The results were expressed as the percentage of HLA-DR-positive monocytes in the total monocyte population.

### Preparation of primary mouse PBMCs

Whole blood from mice was collected by heart puncture bleed into EDTA-K2 vacutainer tubes. PBMCs were separated from whole blood using Ficoll/Paque density gradient centrifugation. The PBMC layer was collected and washed twice with PBS. Total RNA was isolated using TRIzol reagent.

### Plasmid construction

*Renilla* luciferase and firefly luciferase constructs were obtained from Promega (Madison, WI, USA). To create the human TNF-α 3′-untranslated region (UTR)-luciferase reporter construct, the 3′-UTR fragment (789 bp) of TNF-α was cloned into an *Xba*I site located downstream of firefly luciferase in a pGL3-promoter vector. A mutated version (T789 m) of this construct carrying a 7-bp substitution in the miR-181 binding site was obtained through site-directed mutagenesis. The other four truncated TNF-α 3′-UTR luciferase reporter plasmids used in the study were designated T430, T360, T142, and T55. Luciferase constructs with mutations in T55 were designated m1, m2, m3, m4, m5, m6, m7, FM (full mutation), and 181BSM (miR181 binding site mutation), respectively. The human miR-181c/d promoter reporter vector was designated miR-181c/d P1 (CDP1). The vector miR-181c/d P1m (CDP1m) was generated from CDP1 by mutating the three Egr-1 binding sites to “ATCATAATC”. The vector miR-181c/d P2 (CDP2) was created from CDP1 by deleting the 450-bp upstream sequence containing the three Egr-1 binding sites. The coding sequence of human HuR was cloned into a FLAG-tagged pRK5 mammalian expression vector. All primer pairs are provided in Supplementary Table S2.

### Transfections

For knockdown studies, cells were transfected with pools of scrambled or target gene-specific siRNAs at 100 nM (final concentration) using Lipofectamine 2000 according to the manufacturer's instructions. For miRNA studies, cells were transfected with 50 nM miRNA mimics or 200 nM miRNA inhibitors (2′-*O*-methyl antisense oligonucleotides) from RiboBio (Guangzhou, China). THP1 cells were transfected as previously described (Temmerman *et al*, 2012). The siRNA sequences are listed in Supplementary Table S3.

### miRNA and mRNA expression

Total RNA was isolated from intact cells and tissues using TRIzol reagent. Equal amounts of RNA (22 ng for miRNA and 500 ng for mRNA) were used for reverse transcription according to the manufacturer's instructions (Toyobo, Osaka, Japan). For miRNA analysis, the stem-loop RT–PCR method was used as previously described (Chen *et al*, 2005), with minor modifications. Briefly, the RNA was pre-incubated at 65°C for 5 min. The reverse transcriptase reactions (20 μl) contained RNA, 1× RT buffer, RNase-free H_2_O, reverse transcriptase, and 50 nM miRNA-specific stem-loop RT primers. The mix was incubated at 16°C for 10 min, 42°C for 40 min, and 98°C for 5 min. Subsequently, real-time quantification was performed using a StepOnePlus Real-Time PCR system (Applied Biosystems). The relative expression of miRNA was calculated using the 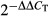 cycle threshold method after normalization to endogenous U6 small RNA (as an internal control). To analyze the expression of target gene mRNA, real-time RT–PCR was performed in the standard way. The mRNA level of each target gene was normalized to the β-actin mRNA level and calculated using the 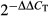 cycle threshold method. GAPDH mRNA was also amplified as an internal control. For semi-quantitative PCR, RNA was reverse transcribed and amplified for 20–30 cycles using gene-specific primers. The primer pairs are listed in Supplementary Table S4.

### CLP and the “two-hit” sepsis model

Male C57BL/6 mice, 6–8 weeks of age, were kept in standard animal house conditions. The animals were anesthetized by i.p. injection with 200–250 μl of 4% chloral hydrate. A 1-cm midline incision was made in the ventral surface of the abdomen. The cecum was exteriorized, and the distal end (about 50%) was ligated with a 3–0 silk suture and punctured once or twice with a 20-gauge needle. In a sham group, a laparotomy was performed in which the cecum was manipulated but not ligated or punctured. The wound was sutured in two layers (muscular and dermal) with 3–0 black silk. For bacterial infections (Echtenacher *et al*, 2003), *Salmonella enterica* Serovar *typhimurium* (*S.tm*.) (ATCC 14028s) was purchased from ATCC. The bacteria were cultured in tryptic soy broth. The number of colony-forming units (CFU) injected was estimated from the optical density at 600 nm and checked by plating aliquots of the inoculum. Mice were injected intraperitoneally with 2 × 10^3^ *S.tm*. in 100 μl of PBS. Kaplan–Meier survival curves were compared using the log-rank test.

### Bacterial counts

Mice were deeply anesthetized with chloral hydrate. Blood was collected by direct cardiac puncture after sterile preparation. Blood (5 μl) was placed on tryptic soy agar plates. Spleen was harvested using sterile technique, weighed, and homogenized with a sterile tissue homogenizer. We serially diluted the suspension and plated 25 μl on tryptic soy agar plates. Bacteria were counted after incubation at 37°C for 24 h and calculated as CFU per ml blood or per g spleen.

### Cytokine detection

The levels of human or mouse TNF-α in culture supernatants or sera were evaluated using enzyme-linked immunosorbent assays (ELISA; R&D Systems, Minneapolis, MN, USA). ELISA was performed according to the manufacturer's instructions.

### Flow cytometric analysis

Spleens or lymph nodes were harvested at the time of sacrifice from all groups of mice. The cells were suspended by gentle grinding and passed through a 70-μm nylon Falcon cell strainer (Becton Dickinson and Co., Franklin Lakes, NJ, USA) to remove connective tissue and large cell clumps. Residual red blood cells were lysed by hypotonic lysis in ice-cold ammonium chloride. Cells were then washed twice in PBS. Total cell counts were obtained using the Invitrogen Countess™ Automated Cell Counter (Invitrogen). Cell viability, assessed with trypan blue, was > 90%. The cells were resuspended in PBS at a concentration of 5 × 10^6^ cells/ml. The percentage of CD4-positive and CD8-positive lymphocytes was determined by simultaneously staining the cells with FITC-CD4 and PE-cy5-CD8a (BD Pharmingen, San Diego, CA, USA). Flow cytometric analysis was performed on a FACScan system (BD Biosciences). Total cell counts were multiplied by the CD4-positive or CD8-positive percentage from the flow cytometer to determine the absolute number of CD4 or CD8 cells.

### Induction of endotoxin tolerance

The cell model of LPS tolerance was adapted from previously described methods (LaRue & McCall, 1994). Human blood monocytes were treated with 1 μg/ml of LPS for 16 h at 37°C in a 5% CO_2_ incubator. After two washes in PBS, the cells were treated with 1 μg/ml LPS for 2 h. Total RNA was extracted using an RNeasy kit (Invitrogen).

### Chromatin immunoprecipitation assay

The ChIP experiments were performed according to the manufacturer's instructions (Millipore, USA). Cells were treated with or without 1 μg/ml LPS for the indicated times, cross-linked with formaldehyde, lysed, and sonicated. Soluble chromatin was incubated overnight with monoclonal anti-human RNA polymerase II antibody (Santa Cruz Biotechnology, Santa Cruz, CA, USA), monoclonal anti-human Egr-1 (15F7) antibody, or rabbit IgG (Cell Signaling Technology, Danvers, MA, USA) as a negative control. The chromatin was then incubated with magnetic protein A beads with rotation for 2 h at 4°C. The protein–DNA cross-linking in the immune complexes was reversed and digested with proteinase K and RNase A. The purified DNA was dissolved with 20 μl of dH_2_O. The human miR-181c/d promoter sequences containing the Egr-1 binding sites were amplified by PCR using the following primers: (forward) 5′-ACAGGTCAAAAGCGACGGGG-3′ and (reverse) 5′-TCACGGTGGTGGCAACGGAA-3′. The control primers were (forward) 5′-AACCCAGGAGGTGGAAAT-3′ and (reverse) 5′-AGCCCAGAAGTTGAGACT-3′. Genomic DNA input was used as an internal standard.

### HuR immunoprecipitation

HuR immunoprecipitation experiments were performed according to the manufacturer's instructions with modifications (Millipore, USA). Briefly, lysates were prepared from A549 cells treated with 100 nM ouabain for the indicated times. Equal amounts of protein lysate were used. HuR polyclonal antibody (Millipore) or isotype control IgG was pre-coated onto magnetic protein A/G beads and extensively washed. Lysates were pre-cleared with IgG. Individual pull-downs were performed at 4°C for 2–3 h to minimize potential reassortment of mRNA. Aliquots of the bound protein complexes were used for SDS–PAGE protein analysis, and the remainder was used for RNA isolation and subsequent mRNA analysis. Immunoprecipitation of HuR-associated β-actin RNA was verified by Q-PCR to confirm the success of the RNA pull-down experiments. The primers used were human β-actin (forward) 5′-TTGTTACAGGAAGTCCCTTGCC-3′ and (reverse) 5′-ATGCTATCACCTCCCCTGTGTG-3′; human TNF-α (forward) 5′-CAGACTTC CTTGAGACACGG-3′ and (reverse) 5′-CAAGGCAGCTCCTACATTGG-3′.

### Indirect immunofluorescence microscopy

Immunofluorescence was performed according to a previously described protocol with minor modifications (Feng *et al*, 2011). Briefly, cells were fixed in 4% formaldehyde and incubated at 37°C with mouse anti-HuR IgG or mouse anti-TIA-1 IgG (Santa Cruz Biotechnology) diluted 1:300 in blocking buffer. A secondary antibody conjugated to an Alexa Fluor dye with the appropriate absorption maximum (488/594) was added for 1–2 h at room temperature. The slides were rinsed and incubated with DAPI (Sigma) for 3 min to stain nuclei. Images were acquired using an Axio Observer microscope (Zeiss) with AxioVision 4.8 Zeiss image-processing software. Images were processed using Photoshop software (Adobe, San Jose, CA, USA).

### RNA fluorescence *in situ* hybridization (RNA-FISH)

RNA-FISH experiments were performed according to a previously described protocol with modifications (Gareau *et al*, 2011). A DNA fragment encompassing the TNF-α coding region was amplified by PCR using primers fused with a T3 (TNF-α forward: 5′-AATTAACCCTCACTAAAGAGCCCATGTTGTAGCAAACC-3′) or T7 (TNF-α reverse: 5′-TAATACGACTCACTATAGGGTAGATGGGCTCATACCAGGG-3′) minimal promoter sequence. The amplified fragments were used as templates for *in vitro* transcription to produce either TNF-α antisense RNAs from the T7 promoter or TNF-α sense RNAs from the T3 promoter, using the FISH Tag RNA Green Kit with Alexa Fluor 488 (Invitrogen, Burlington, ON, Canada). The Alexa Fluor 488-conjugated probe was then purified, quantified, denatured, and incubated with fixed and permeabilized cells, pre-hybridized in 50% PBST/50% hybridization buffer (50% formamide, 5× SSC, 1 mM phosphate buffer, pH 7.4, 1× Denhardt's solution, and 160 ng/ml of denatured salmon sperm DNA) at room temperature for 10 min with gentle rocking. After two washes with fresh hybridization buffer for 30 min at 55°C, the probes were added to the hybridization buffer and incubated with the cells for 16–20 h at 55°C. After hybridization, cells were processed for immuno-fluorescence as described above.

### Western blot analysis

Western blot analyses were performed as previously described (Feng *et al*, 2011). The primary antibodies used were rabbit anti-hemagglutinin (HA) IgG, rabbit anti-Egr-1 (15F7) IgG (Cell Signaling Technology), mouse anti-GAPDH IgG, mouse anti-tubulin IgG, mouse anti-HuR (3A2) IgG, and goat anti-TIA-1 IgG (Santa Cruz Biotechnology). The secondary antibodies used were horseradish peroxidase (HRP)-conjugated anti-mouse IgG or anti-rabbit IgG (Santa Cruz Biotechnology).

### Luciferase assays

Cells were harvested at 24 h after transfection and lysed in 1× lysis buffer. Luciferase activities were measured using a TD20/20n luminometer (Turner BioSystems, Sunnyvale, CA, USA) and a Dual-Luciferase Reporter Assay System (Promega, Madison, WI, USA) according to the manufacturers' instructions. The firefly to *Renilla* luciferase ratio was determined.

### Lentivirus-mediated delivery of HuR *in vivo*

To generate lentiviruses expressing murine HuR, the HuR cDNA sequence was cloned into a pGCSIL-green fluorescent protein (GFP) vector (GeneChem, Shanghai, China). Lentiviral vectors and packaging vectors were then transfected into 293T cells. Supernatants containing lentiviruses were harvested 72 h after transfection and purified through ultracentrifugation. The lentivirus titer was determined using fluorescence-activated cell sorter (FACS) analysis for GFP expression (BD Biosciences). After CLP and *S.tm*. infection for 48 h, mice were challenged with concentrated virus (10^8^ transducing units per mouse) or PBS via the tail vein. Animal survival and bacterial loads in the spleen were examined.

### Statistical analysis

Data are presented in the figures as the mean ± SD. For every figure, statistical tests are justified as appropriate. The significance of the difference between two groups was determined with a two-tailed Student's *t*-test. Multiple comparisons were made with analysis of variance (ANOVA) followed by Bonferroni's *post hoc* test. Survival curve comparisons were performed using a Mantel-Cox log-rank test. For all statistical analyses, GraphPad Prism 5 software for Windows was used (GraphPad Software, San Diego, CA, USA).

## The paper explained

### Problem

Severe sepsis is a serious medical condition normally characterized as an uncontrolled systematic inflammatory response. However, clinical therapies targeting inflammatory cytokines have shown no benefit or, in some cases, have worsened survival. Recent evidence suggests that sepsis-induced immunoparalysis constitutes a major pathogenic mechanism in sepsis and leads to significant morbidity and mortality in critically ill patients. Unfortunately, the effective therapies against sepsis-induced immunoparalysis are lacking.

### Results

We found here that ouabain, as a Na^+^,K^+^-ATPase ligand, was able to reverse sepsis-induced immunoparalysis *in vitro*, *in vivo*, and in clinical samples. The effect of ouabain was critically dependent on the reprogramming of T_H_1 cytokines expression in monocytes at post-transcriptional level, including TNF-α, GM-CSF, and interferon-γ. Ouabain produced two opposite effects on the mRNA stability of TNF-α mRNA mediated by HuR and miR181d. As a result, ouabain-induced HuR nuclear export competed with miR181d for binding to TNF-α mRNA, thereby leading to TNF-α mRNA stabilization and improvement of immunoparalysis.

### Impact

Modulation of TH_1_ cytokines expression at post-transcriptional level could be a useful approach for the therapy of sepsis-induced immunoparalysis. Meanwhile, ouabain is the first identified small molecule drug that can reverse sepsis-induced immunoparalysis. Besides their beneficial effects on the therapy of cardiovascular diseases, cardiac glycosides are also very promising agents for sepsis therapy that deserve further investigation.

## For more information

http://www.nlm.nih.gov/medlineplus/sepsis.html

http://www.survivingsepsis.org/Pages/default.aspx

http://www.sccm.org/Pages/default.aspx

http://www.nigms.nih.gov/education/pages/factsheet_sePSIs.aspx

## Supporting Information

Supplementary Information

Review Process File
