# Supplementary material for: Modulation of TNF-α mRNA stability by human antigen R and miR181s in sepsis-induced immunoparalysis
Source: EMBO Mol Med. 2014 Dec 22;7(2):140–57. doi: 10.15252/emmm.201404797 (PMC4328645; doi:10.15252/emmm.201404797)
Supplement: Supplementary file 1 [file emmm0007-0140-sd1.doc]

**Supplementary Information**


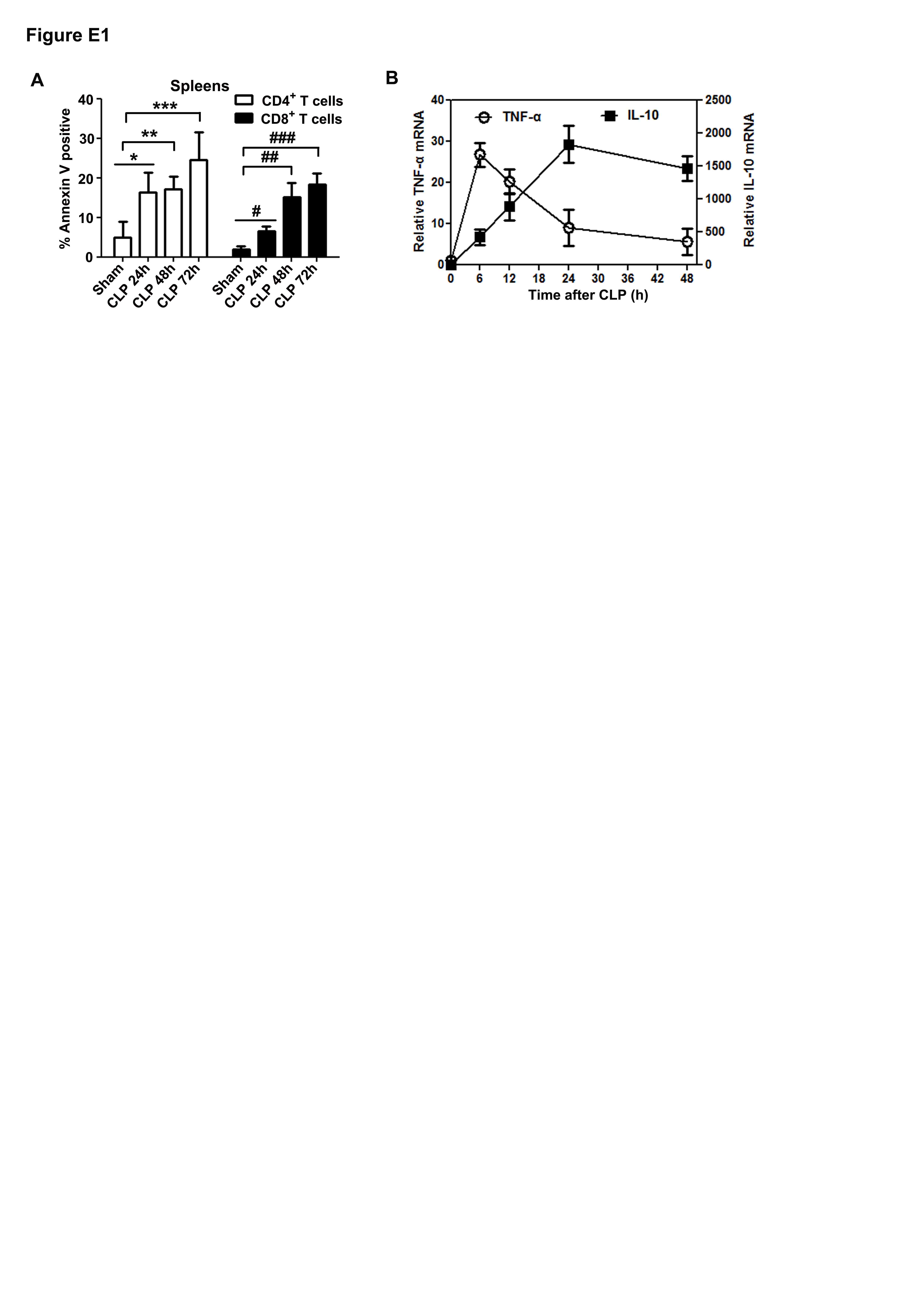


**Supplementary Figure S1.** **The impaired host immunity by cecal ligation and puncture.**

(A) Splenic CD4^+^ and CD8^+^ T-cell apoptosis in mice that underwent CLP. After CLP for 24, 48, and 72 h, Splenic T cells were collected, and stained with CD4^+^, CD8^+^ antibodies and Annexin V-FITC. The results represent mean ± S.D. of three experiments including 6 mice/group. **P* = 0.0366, ***P* = 0.0139, ****P* = 0.0137, ^#^*P* = 0.0064, ^##^*P* = 0.0036, ^###^*P* = 0.0005, as compared to the sham group (Student’s *t*-test).

(B) TNF-α and IL-10 mRNA levels were quantitated by Q-PCR at different time points after CLP. The results represent mean ± S.D. of three experiments including 6 mice/group.

**
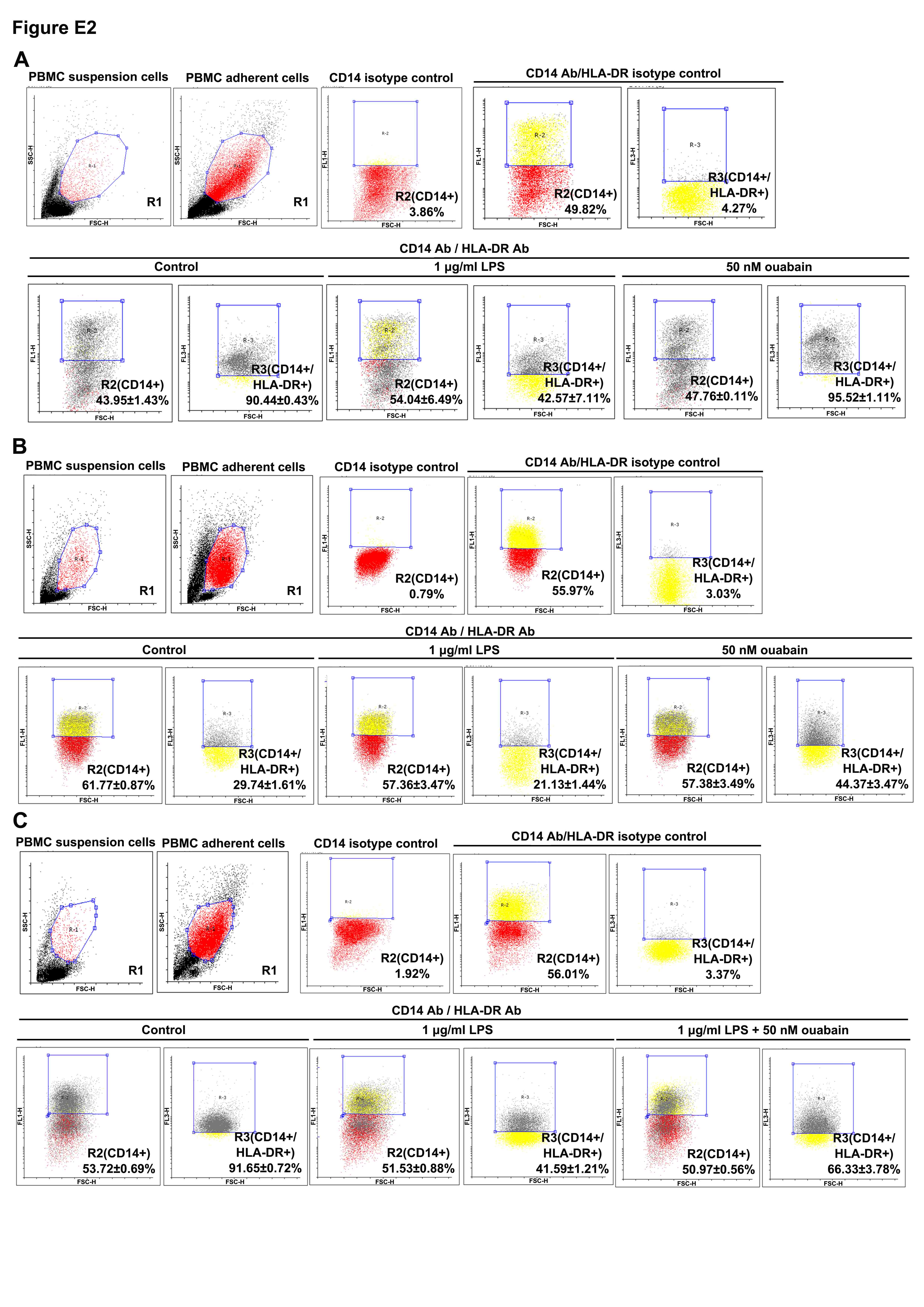
**

**Supplementary Figure S2.** **Flow cytometric analysis of human monocytes HLA-DR expression.**

(A,B) Effects of ouabain and LPS on HLA-DR expression in monocytes from normal donors(A) and sepsis patients (B). The results represent of two independent experiments (8 samples total per condition).

(C) Ouabain reversed LPS-induced suppression of HLA-DR expressions in monocytes from normal donors. The results represent of three independent experiments.

**
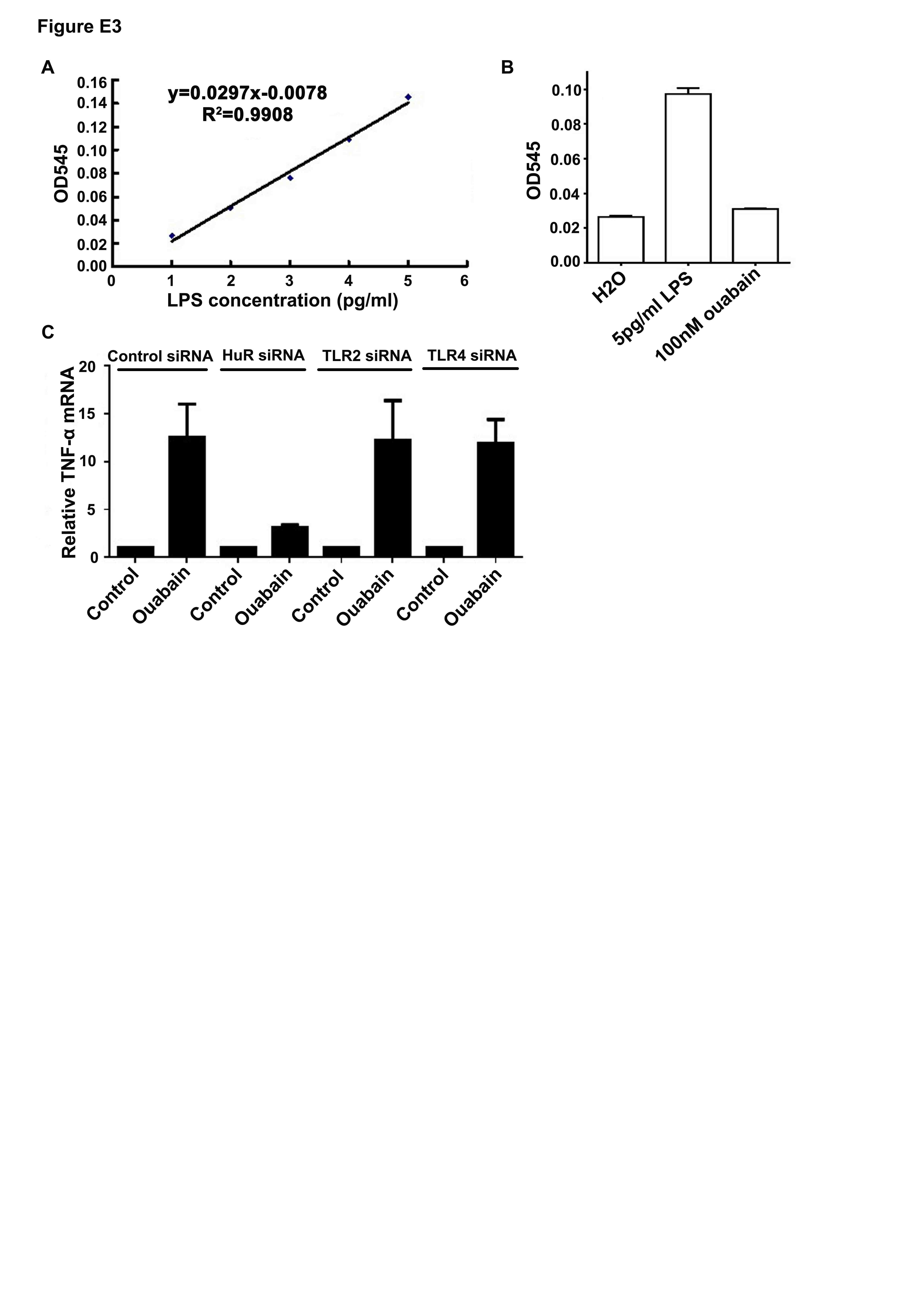
**

**Supplementary Figure S3.** **Measurement of LPS contamination in ouabain solution.**

(A) The standard curve between LPS concentration and OD_545_ value.

(B) The OD_545_ value of H_2_O, 5 pg/ml LPS, and 100 nM ouabain.

(C) Effects of ouabain on TNF-α mRNA expression in A549 cells after transfection with siRNAs against HuR, TLR2, and TLR4, respectively. TLR2 and TLR4 siRNAs are purchased from Santa Cruz biotechnology. After transfection with siRNAs for 24 h, cells with treated with ouabain at 100 nM for 8 h, then total RNA was extracted and Q-PCR was performed to measure TNF-α mRNA level.

**
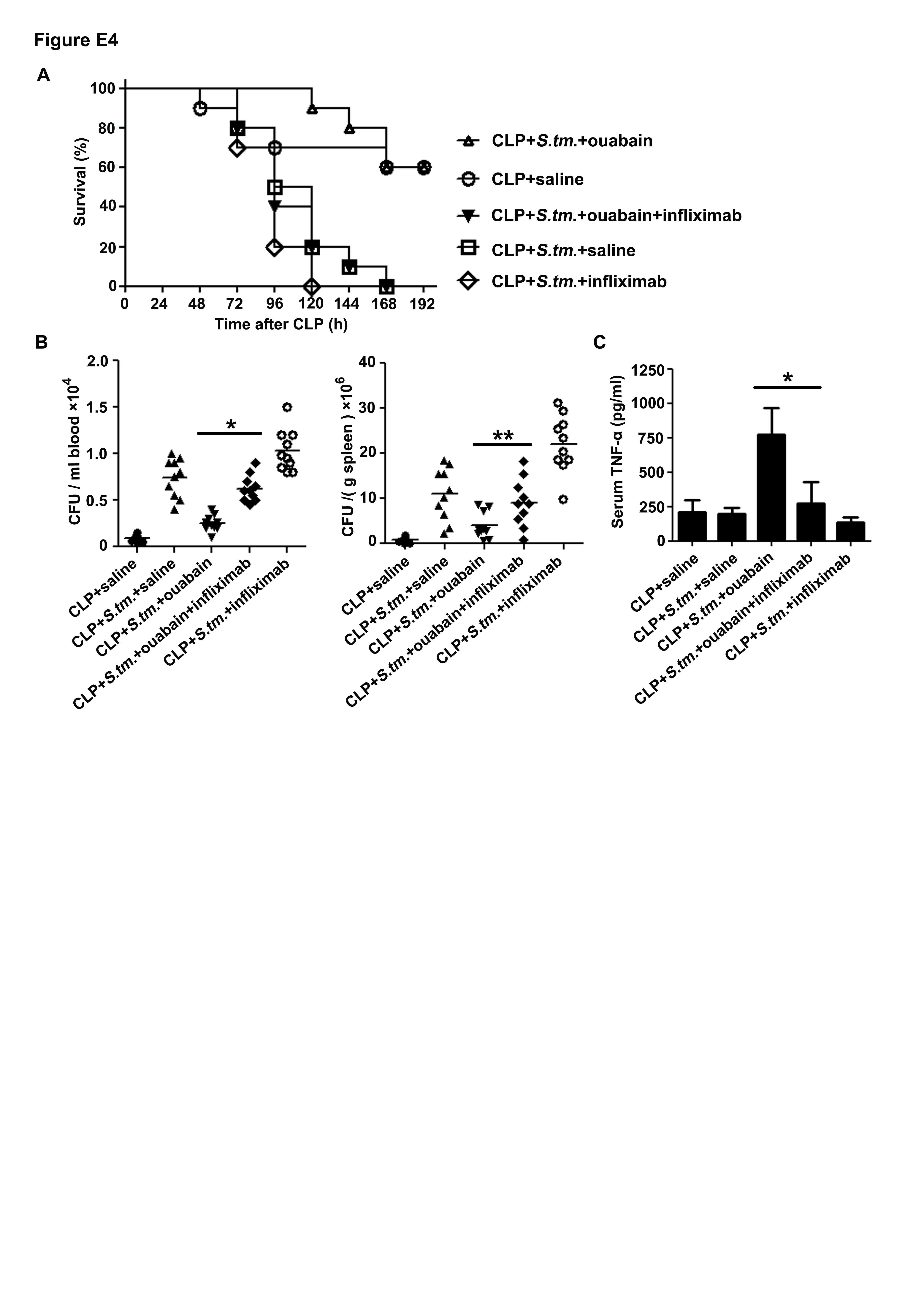
**

**Supplementary Figure S4. Reversal of immunoparalysis by ouabain is dependent on TNF-α.**

(A) Animal survival by ouabain treatment in the CLP with *S.tm.* infection model was blocked by infliximab (300 μg/kg, i.p.). Groups of mice were subjected to moderate CLP (punctured once about 10-20% mortality within 48h), followed 2 days later by infection with *S.tm.* (2×10^3^ CFU, i.p.). After *S.tm.* infection, mice were received saline, ouabain (0.1 mg/kg, i.p.), ouabain with infliximab, or infliximab alone at 54h, 78h and 102h. Survival after CLP and *S.tm.* infection of ouabain-treated versus ouabain with infliximab treated mice was compared (**P* = 0.0002, log-rank test). Data shown are from one experiment (*n* = 12 mice per group), representative of a total of three independent experiments.

(B) Bacterial loads in the blood (left panel) and spleen (right panel) of mice were measured at 48 h after infection with *S.tm.,* as described in the method section. Data shown are from one experiment. (*n* = 10 mice per group), representative of a total of three independent experiments. **P* = 0.00004, ***P* = 0.0275 (one-way ANOVA).

(C) Infliximab neutralized TNF-α protein induced by ouabain. Data shown are the mean of one experiment ± S.D. (*n* = 6-8 mice per group), representative of a total of three independent experiments. **P* = 0.00001 (one-way ANOVA).

**
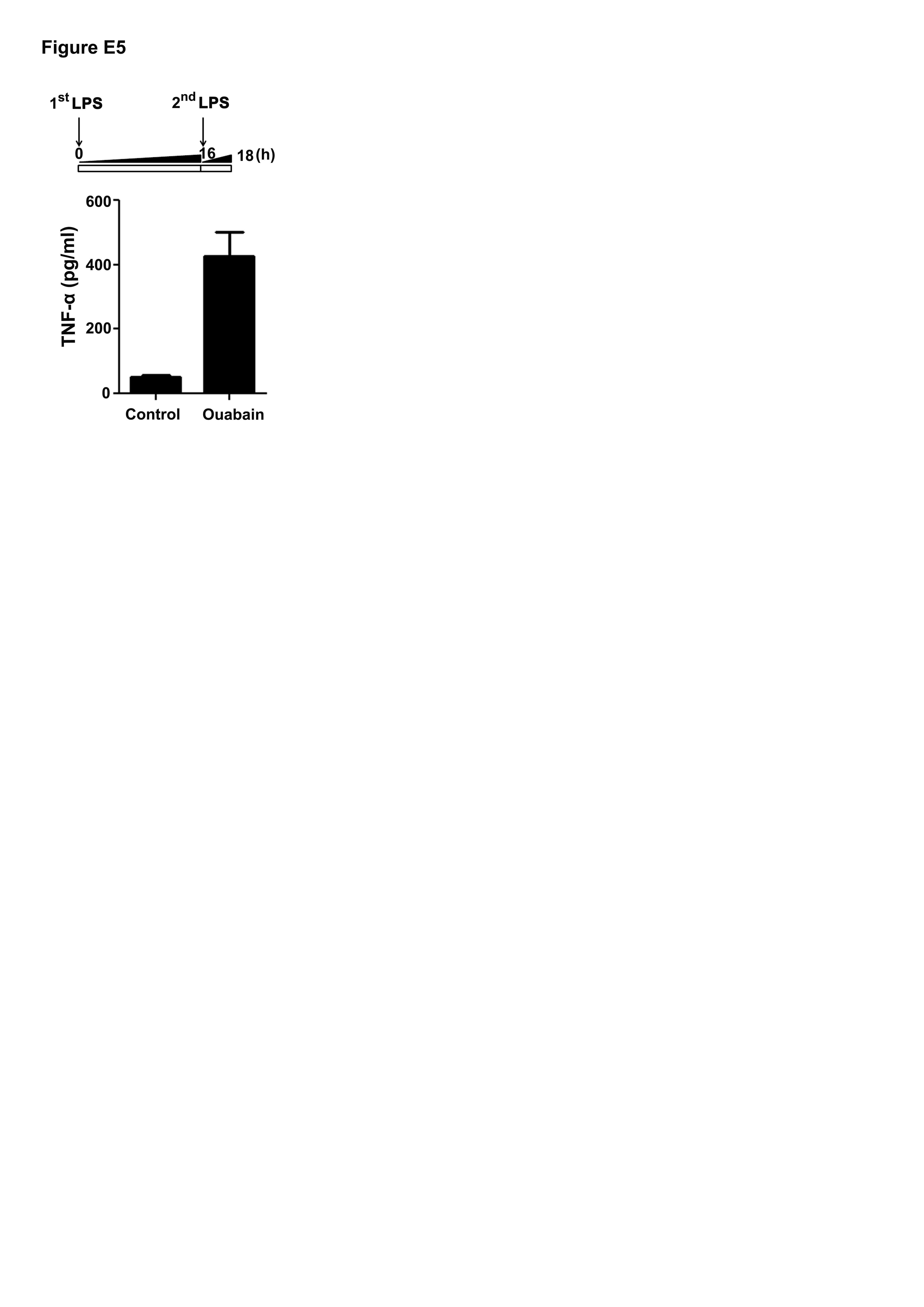
**

**Supplementary Figure S5. Effect of ouabain on LPS-induced endotoxin tolerance.** Monocytes from five healthy volunteer blood were pre-treated with 1 μg/ml LPS only or 1 μg/ml LPS plus 50 nM ouabain for 16 h, and then re-stimulated with 1 μg/ml LPS for 2 h. Supernatant TNF-α levels were determined by ELISA. Data are expressed as mean ± S.D. from three independent experiments.

**
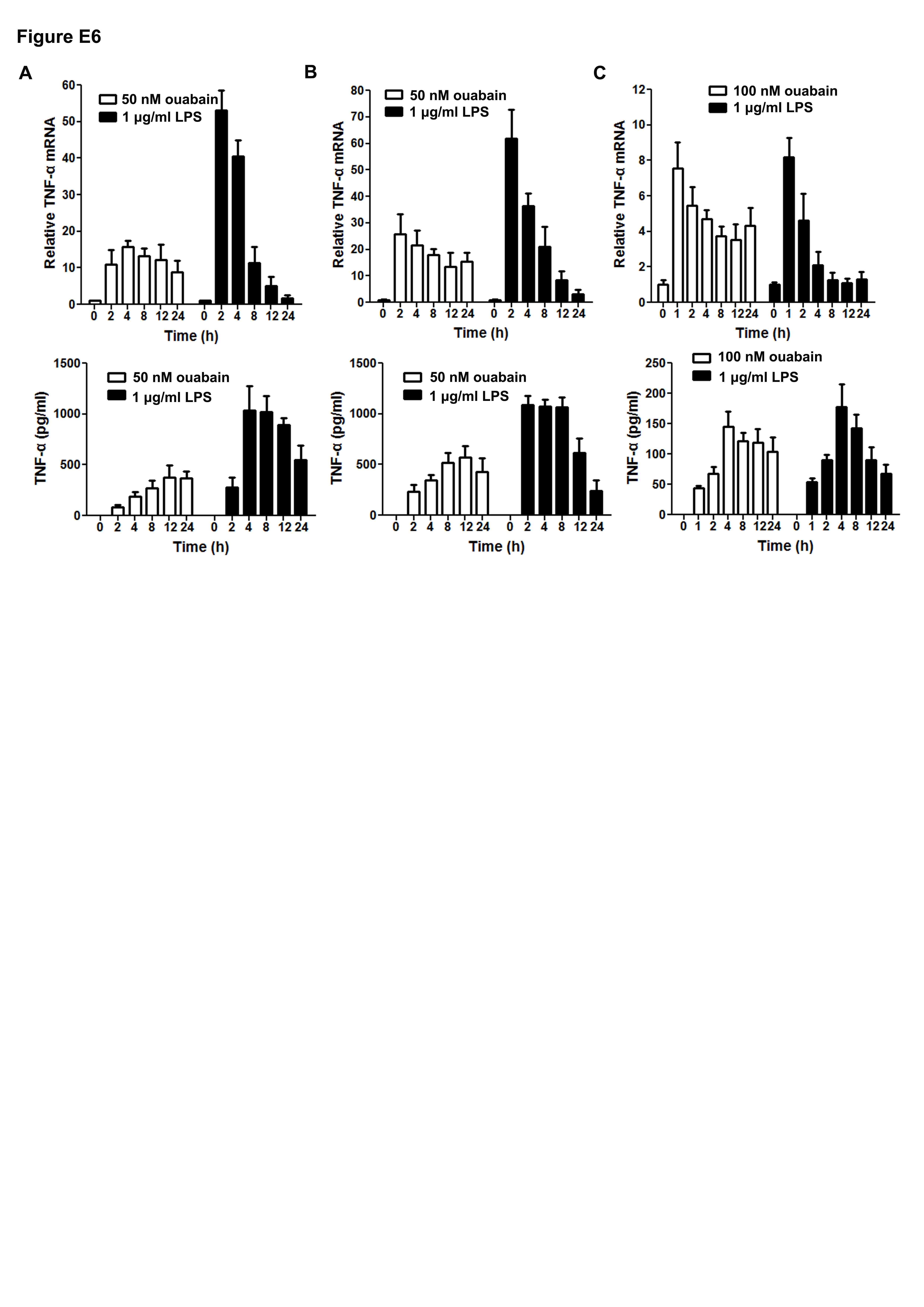
**

**Supplementary Figure S6.** **Time-dependent TNF-α mRNA or protein expression in human blood monocytes (A), THP-1 cells (B), and A549 cells (C) after treatment with ouabain or LPS.** All data are obtained from three independent experiments (mean ± S.D.).


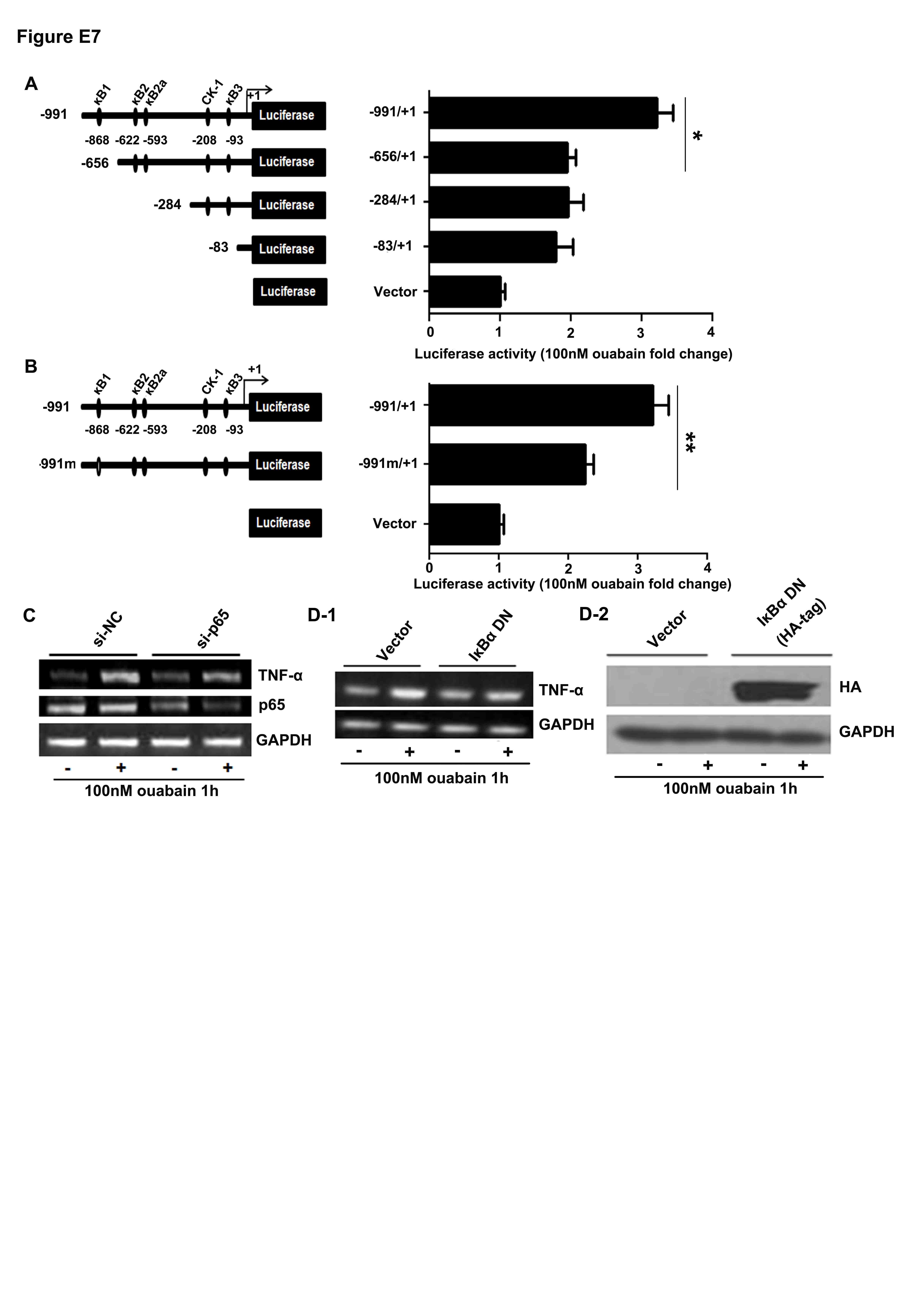


**Supplementary Figure S7. Involvement of NF-κB in the upregulation of TNF-α gene transcription by ouabain.**

(A,B) Identification of ouabain-responsive cis-elements within the TNF-α promoter by sequential deletion (A, left) and sites mutation analysis (B, left). A549 cells were transfected with a series of TNF-α promoter variants, further treated with ouabain for 12 h. The fold increase of luciferase activity (firefly/renilla) of each transfection group after ouabain treatment is indicated (A and B, right). *P= 0.0009, **P= 0.0024 (one-way ANOVA). Data are obtained from three independent experiments (mean ± S.D.).

(C) The p65 silencing suppressed ouabain-induced TNF-α mRNA expression in A549 cells. Cells were transfected with p65 siRNA or control siRNA (100 pmol) for 24 h, and then treated with ouabain at 100 nM for 1h. GAPDH was included as a control.

(D) Overexpression of IκBα DN suppressed ouabain-induced TNF-α mRNA expression in A549 cells. Cells were transiently transfected with 2 μg vector, or IκBα DN overexpressing plasmid, further treated with ouabain at 100 nM for 1h. TNF-α mRNA levels were determined by RT-PCR (D-1). Western-blot analysis was performed to measure IκBα DN (HA-tag) plasmid expression levels (D-2). All experiments were performed at least in triplicate.


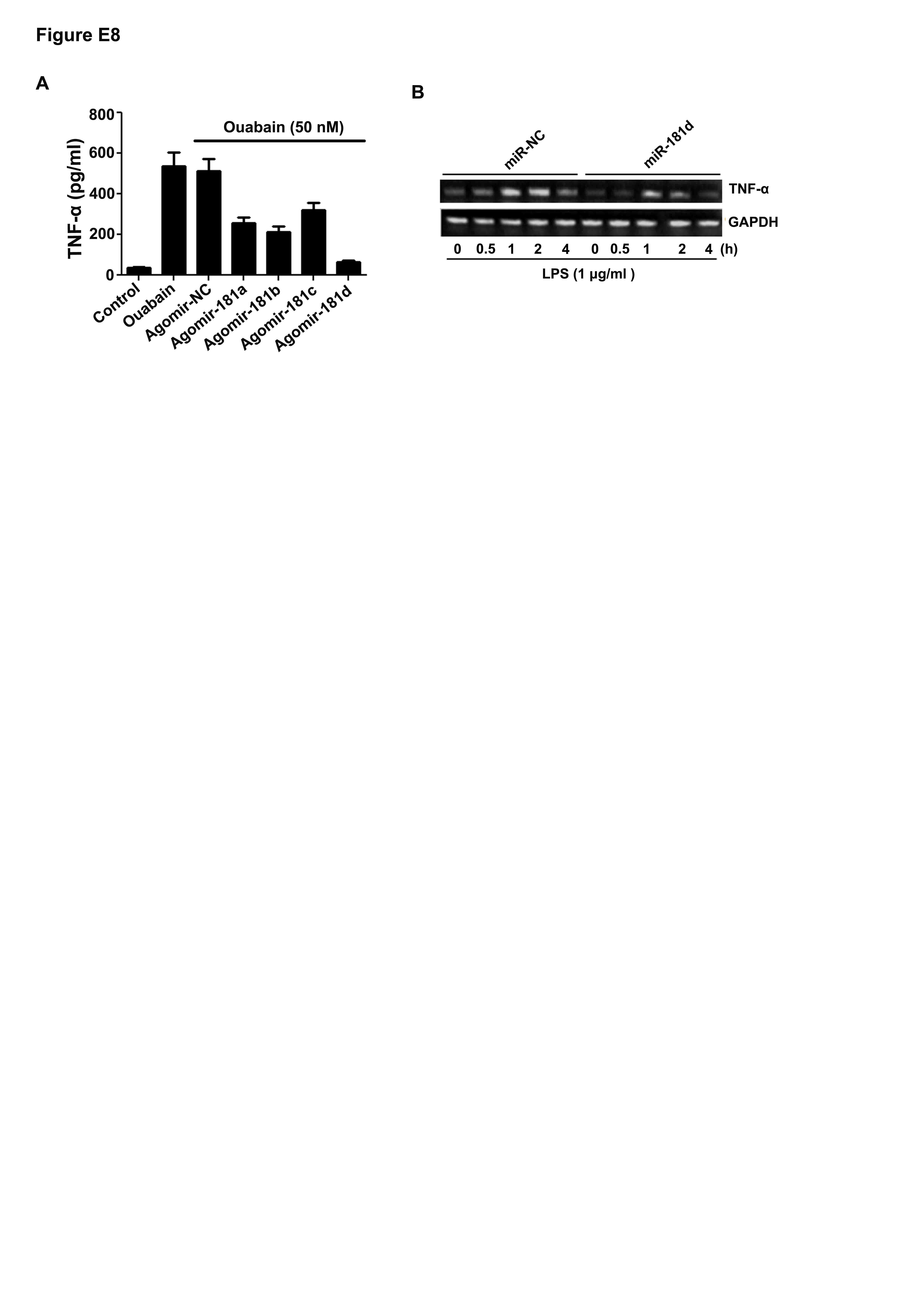


**Supplementary Figure S8.** **Effects of miR-181s on TNF**-α **expressions.**

(A) miR-181s suppressed ouabain-induced TNF-α expression. Monocytes from 5 healthy volunteer were isolated, pre-treated with 400 pmol agomir-181s or matched agomir-NC for 24 h, further treated with 50 nM ouabain for 8 h. Supernatant TNF-α protein levels were determined by ELISA. Data are obtained from two independent experiments.

(B) miR-181d suppressed LPS-induced TNF-α gene transcription. A549 cells were transfected with miR-181d mimics or miR-NC at a final concentration of 50 nM. 24 hours later, these cells were stimulated with LPS (1 μg/ml) for the indicated time. The TNF-α mRNA were measured by RT-PCR and normalized to the expression of GAPDH mRNA in each sample. The experiments were performed in triplicate.

**
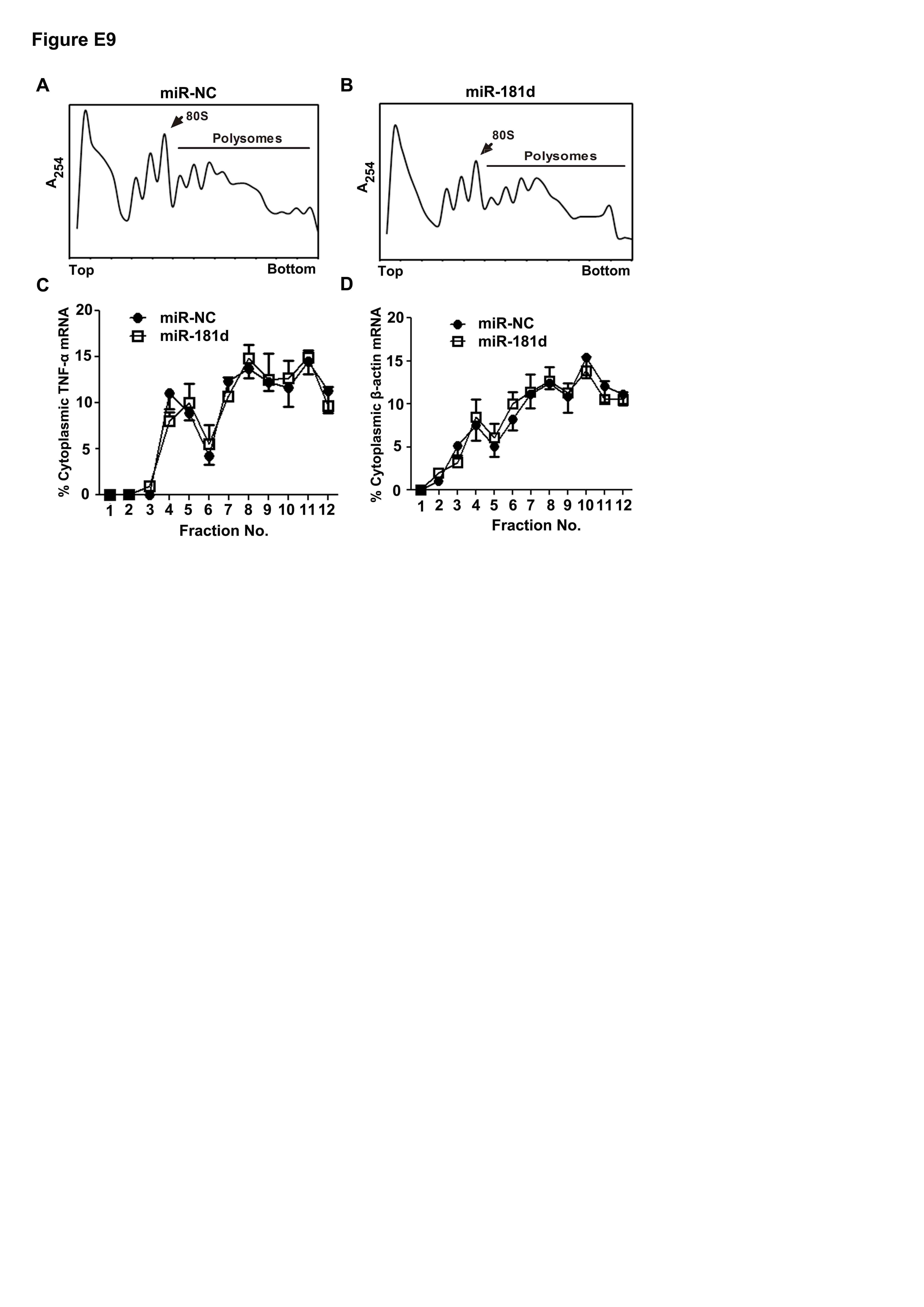
**

**Supplementary Figure S9. Effect of miR-181d on the translation of TNF-α mRNA.**

A549 cells were transfected for 24 h with either miR-NC (A) or miR-181d (B). Lysates were subjected to sucrose density gradient fractionation and RNA was monitored by absorption at 254nm (top). RNA isolated from each fraction was subjected to Q-PCR to determine TNF-α (C) and β-actin mRNAs (D).

A549 cells were seeded in 15 cm dishes 1 day prior to treatment of the cells. Subsequently, translation was analyzed by polysomal fractionation according to previously described protocol with some changes. Briefly, 100 μg/mL cycloheximide (CHX) was added to cells for the last 10 min of the incubations at 37°C. Then cells were scraped off in PBS/CHX (100 μg /mL) and added in 750 μl polysome buffer (20 mM Tris-HCl (pH 7.5), 140 mM KCl, 5 mM MgCl_2_, 0.3% (v/v) Triton X-100, 100 μg/mL cycloheximide, 10 μM PMSF, 0.5 mg/mL heparin, and 100 U/mL RNasin), then immediately transferred the solution to a 5-ml Dounce homogenizer on ice. Lysed cells were centrifuged at 13,000 g for 10 min at 4°C. Protein concentration of cytoplasmic lysate was measured by Bradford assay. Approximately 2.5 mg cytoplasmic lysate proteins were layered on top of linear 7%–47% (w/v) sucrose gradients (11 ml). The gradients were centrifuged at 39,000 rpm for 2h at 4°C without brake using a SW41 rotor in a Beckman ultracentrifuge. Gradients were collected in 1 mL fractions starting from the top of the gradients. Absorbance was measured at 254 nm. Each fraction was digested with 0.2 mg/ml proteinase K and 1% SDS (final concentration) at 37°C for 30 min, phenol extracted , 1/10 volume sodium acetate (3 M) and 1 volume isopropanol precipitated, 70% ethanol washed and resuspended in 20 μl of DEPC-treated water. The relative amounts of TNF-α and β-actin mRNA in each fraction were determined by Q-PCR. Data shown are the mean of one experiment ± S.D., representative of a total of three independent experiments.


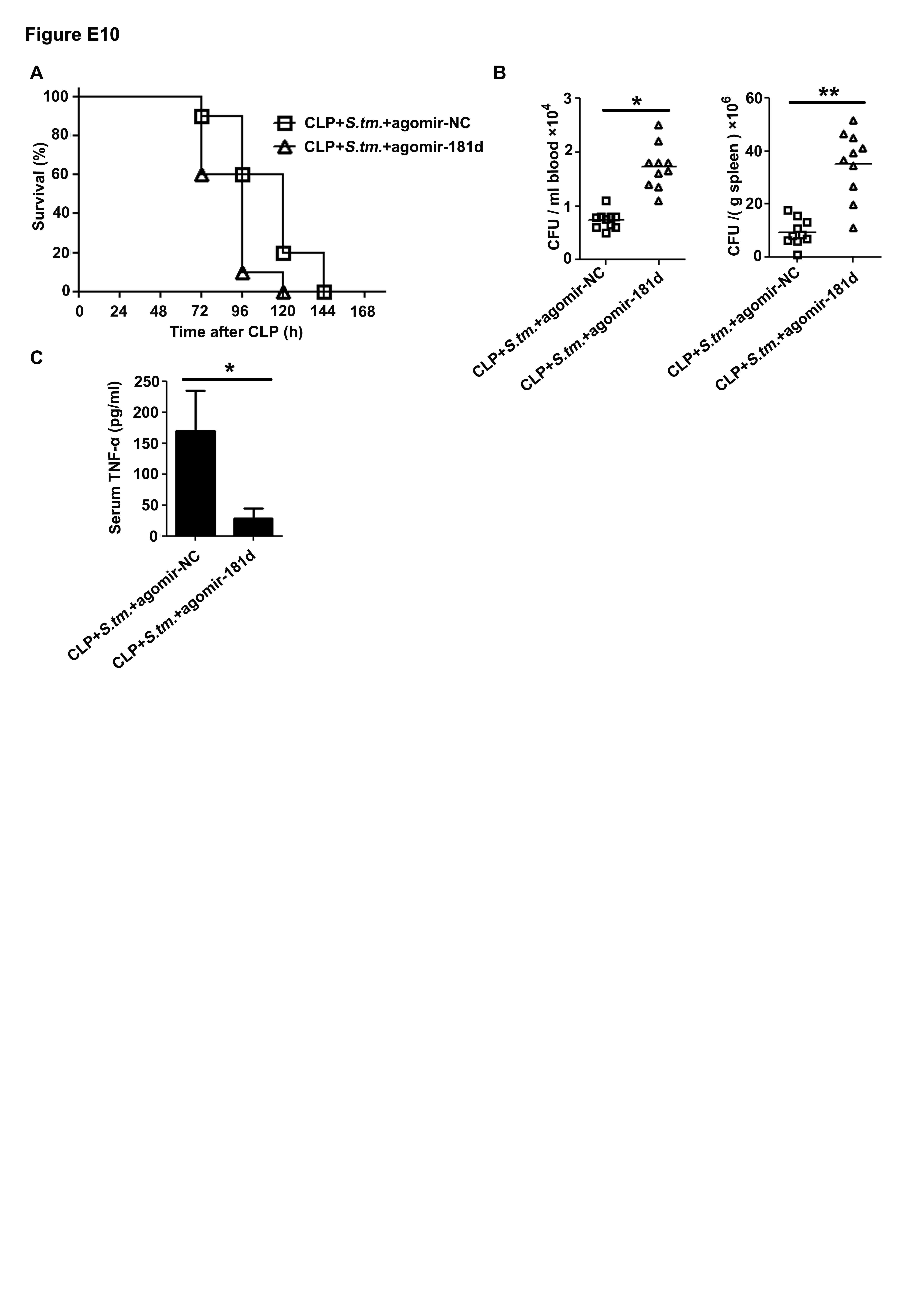


**Supplementary Figure S10.** **Effect of agomir-181d on immunoparalysis.**

(A) Groups of mice were subjected to moderate CLP (punctured once about 10-20% mortality within 48h), followed 2 days later by infection with *S.tm.* (2×10^3^ CFU, i.p.). After *S.tm.* infection, mice were injected with agomir-181d or agomir-NC via tail-vein at the dose of 10 nmol per mouse at 54h, 78h and 102h. Survival after CLP and *S.tm.* infection of agomir-181d -treated versus agomir-NC -treated mice was compared (**P* = 0.0145, log-rank test). Data shown are from one experiment (*n* = 12 mice per group), representative of a total of three independent experiments.

(B) Bacterial loads in the blood (left panel) and spleen (right panel) of mice were measured at 48 h after infection with *S.tm.,* as described in the method section. Data shown are from one experiment (*n* = 10 mice per group), representative of a total of three independent experiments. **P* = 0.000001, ***P* = 0.00001 (Student’s *t*-test).

(C) Agomir-181d reduced the serum TNF-α of CLP mice with *S.tm.* infection. Data shown are the mean of one experiment ± S.D. (*n* = 6-8 mice per group), representative of a total of three independent experiments. **P* = 0.00011 (Student’s *t*-test).

**
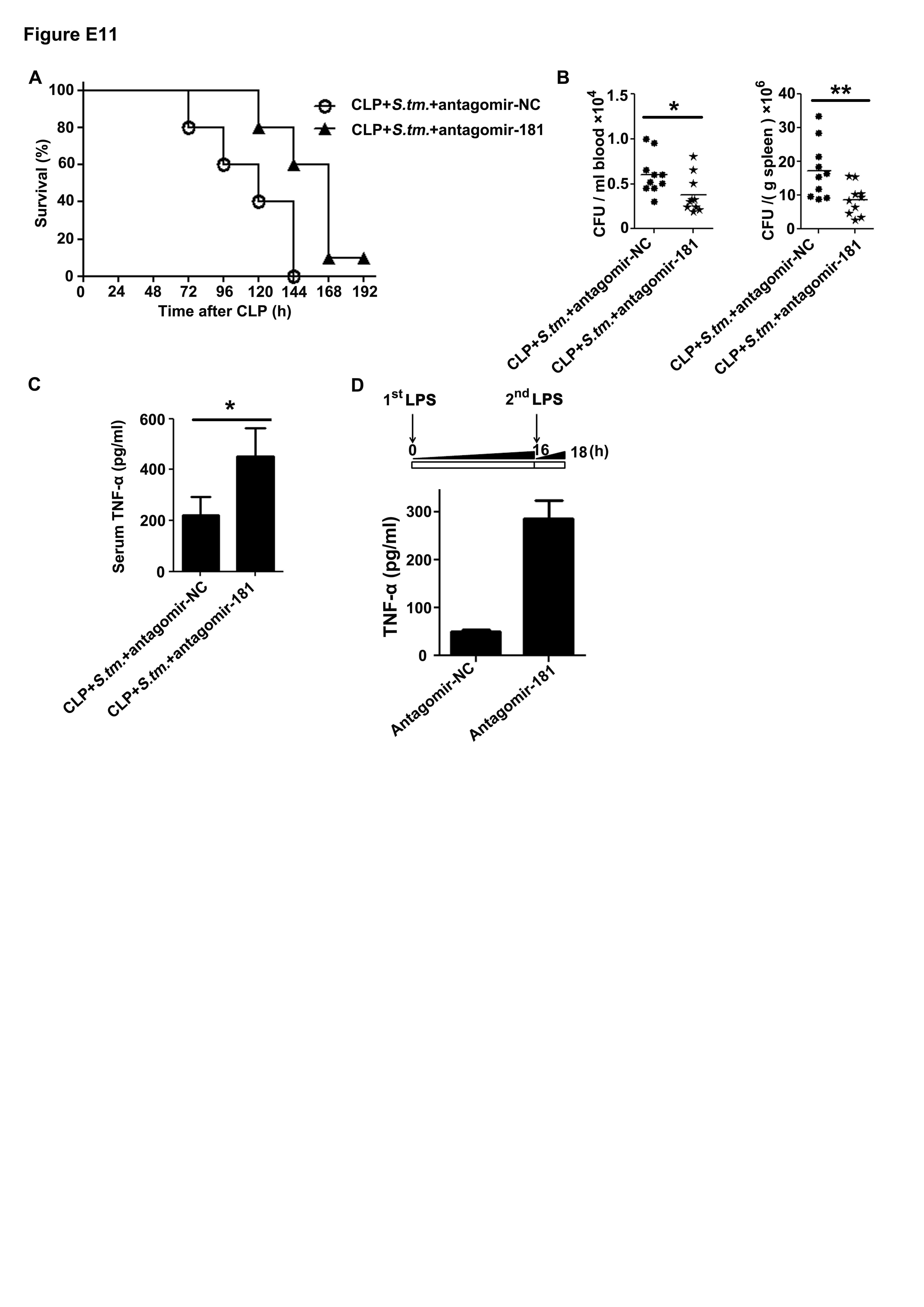
**

**Supplementary Figure S11.** **Effect of antagomir-181s on immunoparalysis.**

(A) Groups of mice were subjected to moderate CLP (punctured once about 10-20% mortality within 48h), followed 2 days later by infection with *S.tm.* (2×10^3^ CFU, i.p.). After *S.tm.* infection, mice were injected with antagomir-181s (including antagomir-181a/b/c/d) or antagomir-NC via tail-vein at the dose of 10 nmol per mouse at 54h, 78h and 102h. Survival after CLP and *S.tm.* infection of agomir-181d -treated versus agomir-NC -treated mice was compared (**P* = 0.003, log-rank test). Data shown are from one experiment (*n* = 12 mice per group), representative of a total of three independent experiments.

(B) Bacterial loads in the blood (left panel) and spleen (right panel) of mice were measured at 48 h after infection with *S.tm.,* as described in the method section. Data shown are from one experiment (*n* = 10 mice per group), representative of a total of three independent experiments. **P* = 0.0297, ***P* = 0.0105 (Student’s *t*-test).

(C) Antagomir-181s induced the serum TNF-α of CLP mice with *S.tm.* infection. Data shown are the mean of one experiment ± S.D. (*n* = 6-8 mice per group), representative of a total of three independent experiments. **P* = 0.0011 (Student’s *t*-test).

(D) Antagomir-181 reversed LPS-induced endotoxin tolerance. Monocytes from 5 healthy volunteer were isolated, pre-treated with 800 pmol antagomir-181 or matched antagomir control for 24 h, further treated with 1 μg/ml LPS for 16 h, and then re-stimulated with 1 μg/ml LPS for 2 h. Supernatant TNF-α protein levels were determined by ELISA. Data represent the mean ± S.D. from two independent experiments.

**
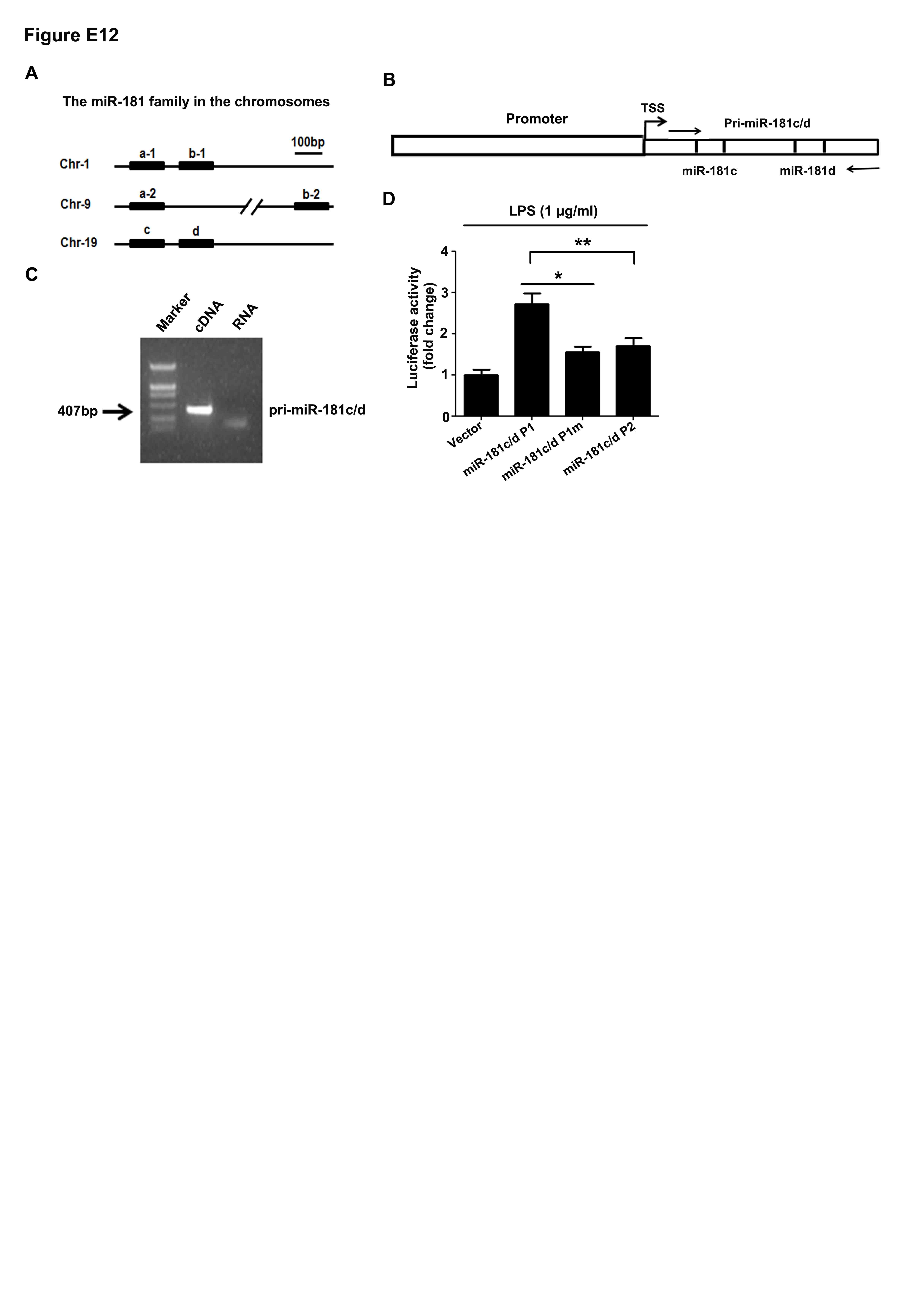
**

**Supplementary Figure S12. Egr-1 is involved in miR-181c/d transcription by ouabain.**

(A) Schematic depiction of human miR-181 family in genome.

(B) Schematic representation of the pri-miR-181c/d sequence and its promoter. The bent arrow indicates the transcription start site (TSS).

(C) RT-PCR analysis was performed to verify the presence of pri-miR-181c/d in A549 cells treated with LPS at a concentration of 1 μg/ml for 2 h, DNA maker is indicated. (D) Effect of LPS on the luciferase activity of miR-181c/d promoter and its mutants. A549 cells were transiently transfected with 1 μg firefly luciferase plasmids and 50 ng pRL-TK, treated with 1 μg/ml LPS for an additional 8 h. The luciferase activity (firefly/renilla) of each transfection group is indicated. The luciferase activity of the vector was arbitrarily set at 1.0. Data represent the mean ± S.D. of three experiments. **P* = 0.00001, ***P* = 0.00003 (one-way ANOVA).


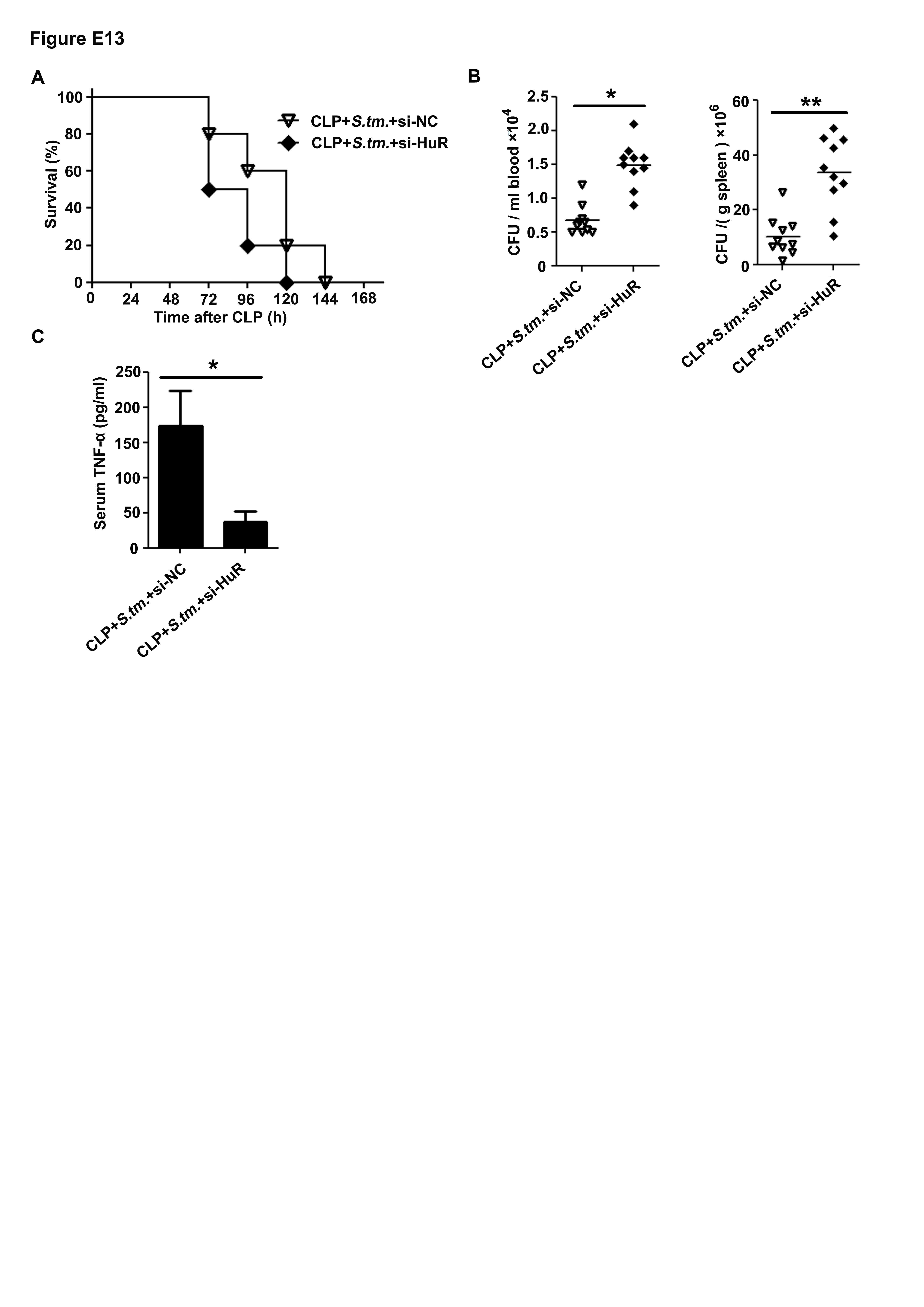


**Supplementary Figure S13. Effect of HuR siRNA on immunoparalysis.**

(A) Groups of mice were subjected to moderate CLP (punctured once about 10-20% mortality within 48h), followed 2 days later by infection with *S.tm.* (2×10^3^ CFU, i.p.). After *S.tm.* infection, mice were injected with cholesterol-modified HuR siRNA or control siRNA via tail-vein at the dose of 10 nmol per mouse at 54h, 78h and 102h. Survival after CLP and *S.tm.* infection of HuR siRNA -treated versus control siRNA -treated mice was compared (**P* = 0.0428, log-rank test). Data shown are from one experiment (*n* = 12 mice per group), representative of a total of three independent experiments.

(B) Bacterial loads in the blood (left panel) and spleen (right panel) of mice were measured at 48 h after infection with *S.tm.,* as described in the method section. Data shown are from one experiment. (*n* = 10 mice per group), representative of a total of three independent experiments. **P* = 0.000004, ***P* = 0.00012 (Student’s *t*-test).

(C) HuR silencing reduced the serum TNF-α of CLP mice with *S.tm.* infection. Data shown are the mean of one experiment ± S.D. (*n* = 6-8 mice per group), representative of a total of three independent experiments. **P* = 0.00002 (Student’s *t*-test).


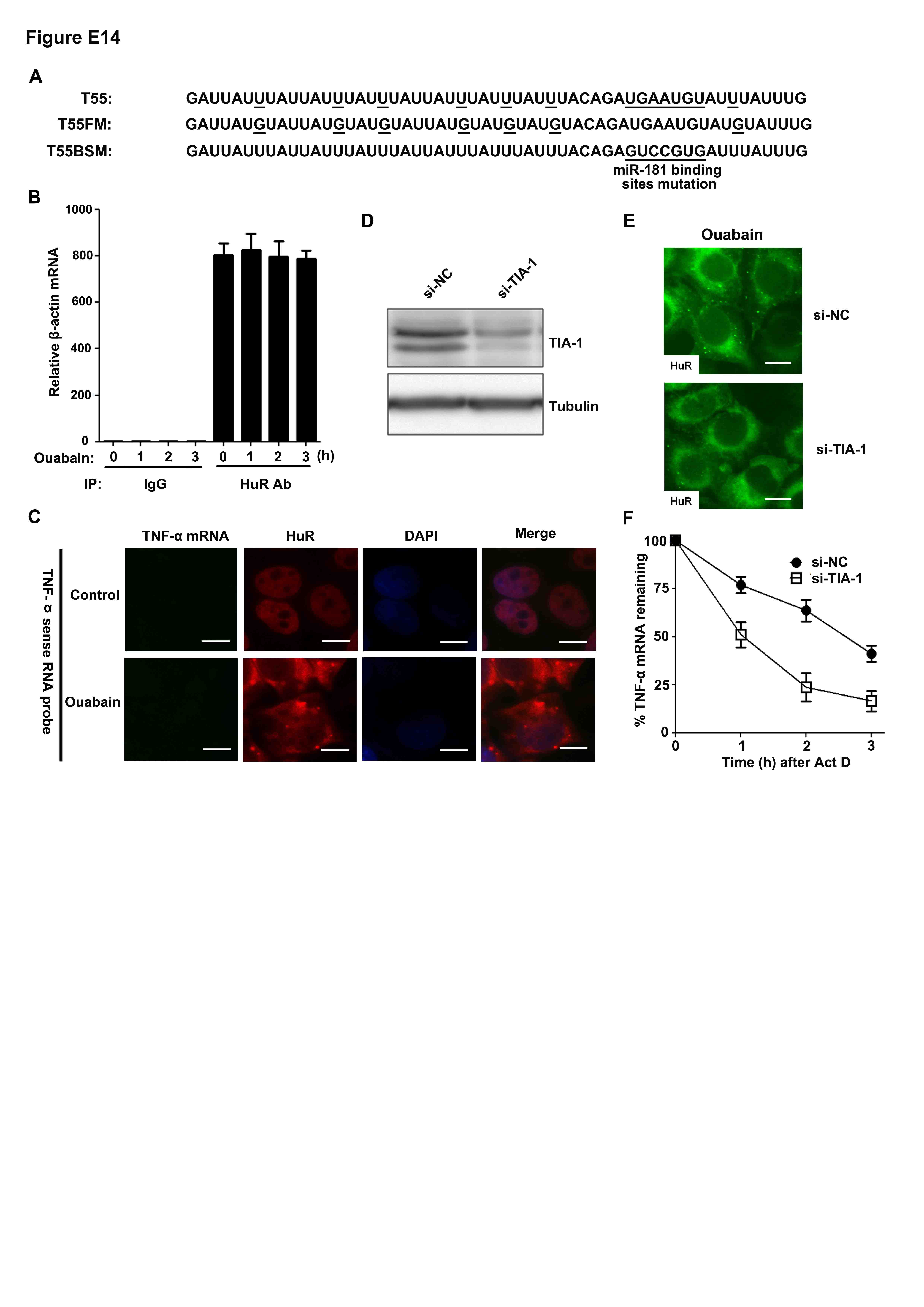


**Supplementary Figure S14. HuR associates with TNF-α mRNA and recruits TNF-α mRNA to stress granules for protection.**

(A) Point mutations of miR-181s and HuR binding sites in T55.

(B) Association of cytoplasmic HuR with β-actin mRNA by RNA immunoprecipitation experiment.

(C) Ouabain recruited TNF-α mRNAs to SGs. After treatment with ouabain (100 nM) for 12 h, A549 cells were fixed, permeabilized, and incubated with 3 nM of an Alexa Fluor 488-labeled sense RNA probe as control. SGs were visualized by counterstaining for HuR, as indicated in red color. DAPI was used for staining nuclei. Merged views are shown in the right panels. Scale bars: 10 μm. Shown are typical results from 5 different fields and 3 different experiments.

(D-1) Western-blot analysis was performed to measure the level of TIA-1 after silencing. Data shown are from one experiment, representative of a total of three independent experiments.

(D-2) Silencing of TIA-1 expression suppressed ouabain-induced SGs assembly. Scale bars: 10 μm. Representative fluorescent images of 3 independent experiments showing similar results are shown.

(D-3) Silencing of TIA-1expression shortened the half life of TNF-α mRNA. Data are from 3 independent experiments (mean ± S.D.).

**
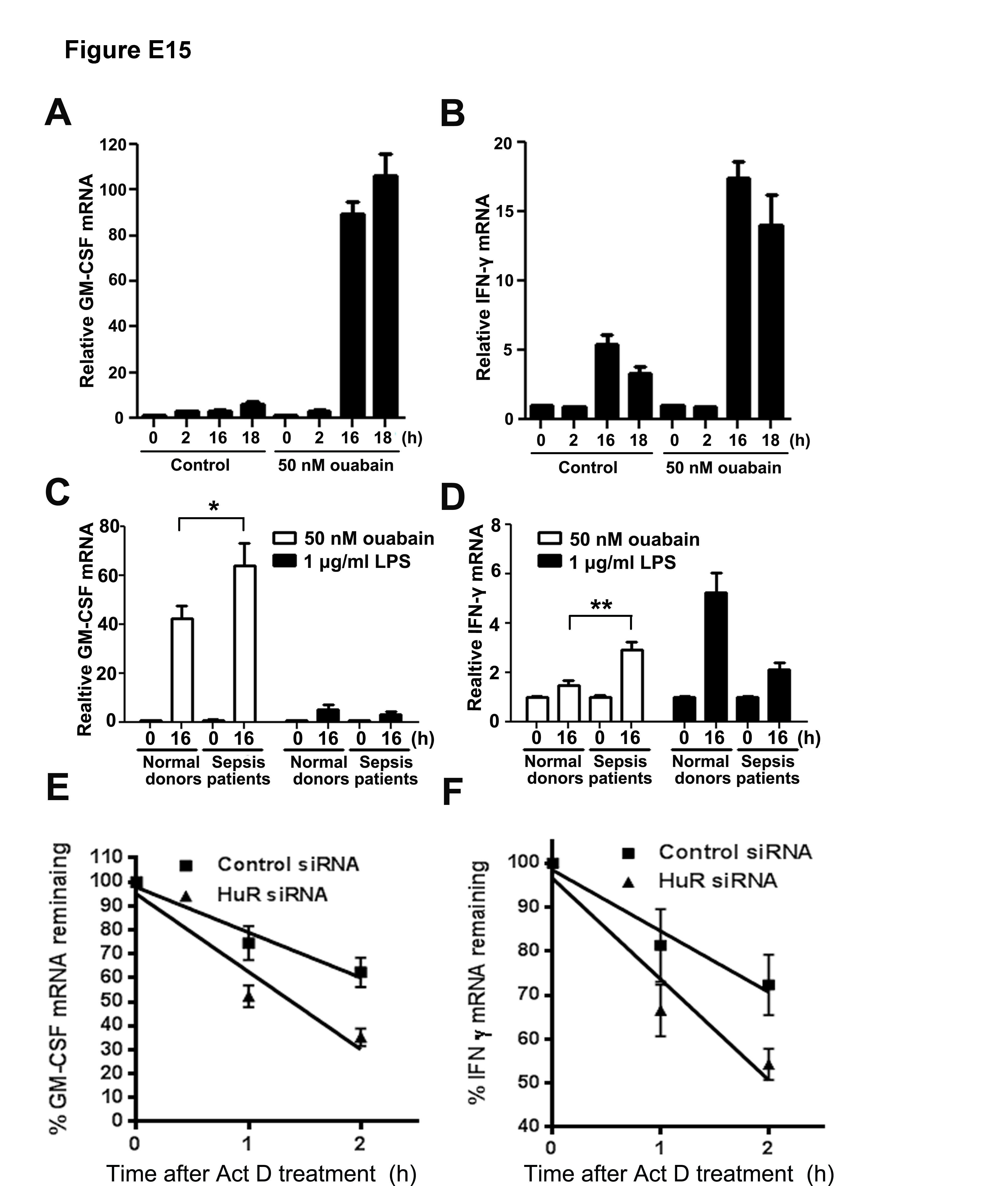
**

**Supplementary Figure S15. Effect of ouabain on GM-CSF and IFN-γ mRNA expression and stability.**

(A, B) Five healthy volunteer blood monocytes were treated with 1 μg/ml LPS alone or LPS plus 50 nM ouabain for 16 h, then re-stimulated with 1 μg/ml LPS for 2 h. The mRNA expression levels of GM-CSF and IFN-γ were measured by Q-PCR.

(C, D) Heparinized blood was obtained from 5 healthy volunteer donors and 5 severe sepsis patients. Isolated monocytes were treated with 1μg/ml LPS or 50nM ouabain for the indicated time. (*A, C*) GM-CSF and (*B, D*) IFN-γ mRNA levels were determined by Q-PCR. Sample data were normalized to β-actin mRNA. **P* = 0.0247, ***P* = 0.0022 (Student’s *t*-test). Data represent the mean ± S.D. from 2 independent experiments and are presented as fold change relative to unstimulated cells (set at 1-fold).

(E,F) Silencing of HuR shortened the half life of GM-CSF (*E*) and IFN γ (*F*) mRNAs. Human monocytes isolated from normal donors were introduced with cholesterol-modified HuR siRNA or control siRNA (100 pmol) for 24 h, treated with ouabain at 200 nM for 1 h before 5 μg/ml Act D was added for the indicated time, then harvested and extracted for total cellular RNA. At the indicated time, GM-CSF and IFN γ mRNA levels were quantified by Q-PCR by using GAPDH as normalization control. Data are from 3 independent experiments (mean ± S.D.).


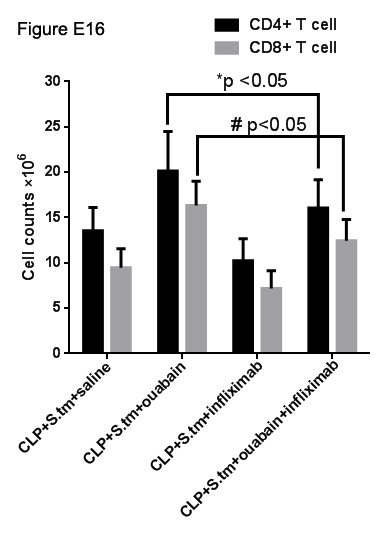

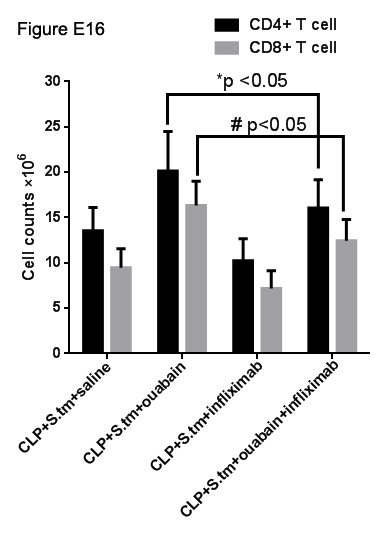


**Supplementary Figure S16. Effect of infliximab on ouabain-induced increases in splenic CD4^+^ and CD8^+^ T cells in “two-hit” sepsis model**. Splenic CD4+ and CD8+ T cells were calculated at 48 h after infection with S.tm., as described in the method section. **P <0.05, # P<0.05* (one-way ANOVA).

**
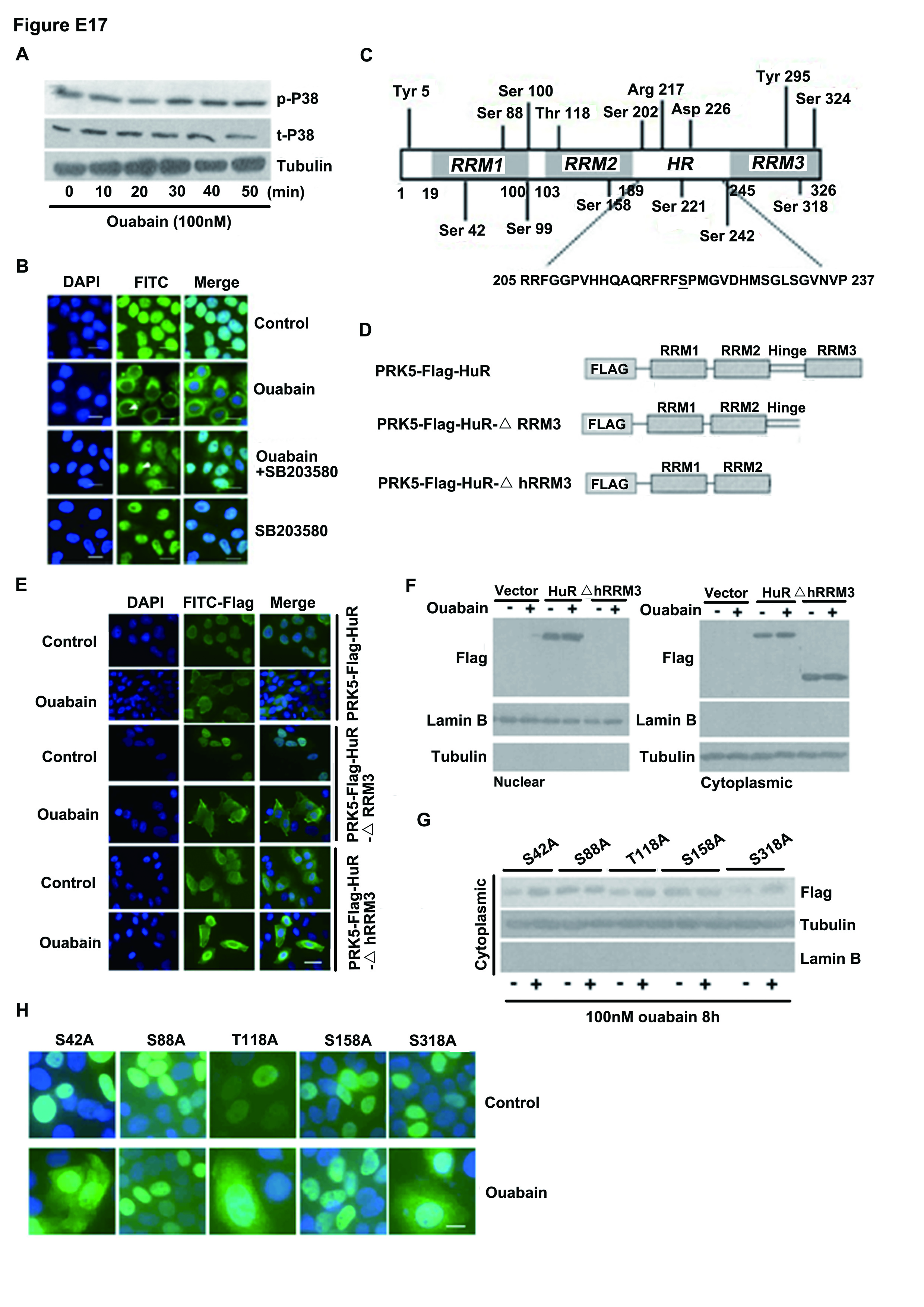
**

**Supplementary Figure S17. Involvement of p38 signaling in ouabain-induced HuR nuclear export.** (A) A549 cells were treated with ouabain at 100 nM for 0, 10, 20, 30, 40, and 50 min respectively. After treatments, cells were collected and subject to western blot analysis by using the indicated IgGs.

(B) Cells were pretreated with 20 μM SB203580 for 30 min, the treated with ouabain at 100 nM for 8 h, cells after treatment were fixed, stained with HuR IgG, and then FITC-conjugated secondary IgG. After treatment, cells were observed under fluorescence microscope, scale bars: 20 μm.

© Schematic diagram of HuR phosphorylation sites.

(D) Construction of HuR variants short of RRM3 motif, or both RRM3 motif and hinge region.

(E) A549 cells were transfected with FLAG-tagged HuR or its variants for 24 h, the treated with ouabain at 100 nM for an additional 8 h. After treatments, cells were fixed, stained with HuR IgG and FITC-conjugated secondary IgG, and then observed under fluorescence microscope.

(F) A549 cells were transfected with FLAG-tagged HuR or its variants, proteins of nuclear and cytoplasm fractions were extracted and subjected to western blot analysis. Lamin B and tubulin were used as loading controls for the nuclear and cytoplasm fractions respectively.

(G) A549 cells were transfected with HuR point mutations, S42A, S88A, T118A, S158A, and S318A respectively for 24 h, after transfection, cells were treated with ouabain at 100 nM for 8 h, cytoplasm fractions were extracted and subject to western blot analysis by using the indicated IgGs.

(H) Cells after transfection with HuR point mutations were probed with HuR IgG and FITC-conjugated secondary IgG, and then observed under fluorescent microscope.

**Supplementary Table S1. Characteristics of thirty-three patients with severe sepsis**

**
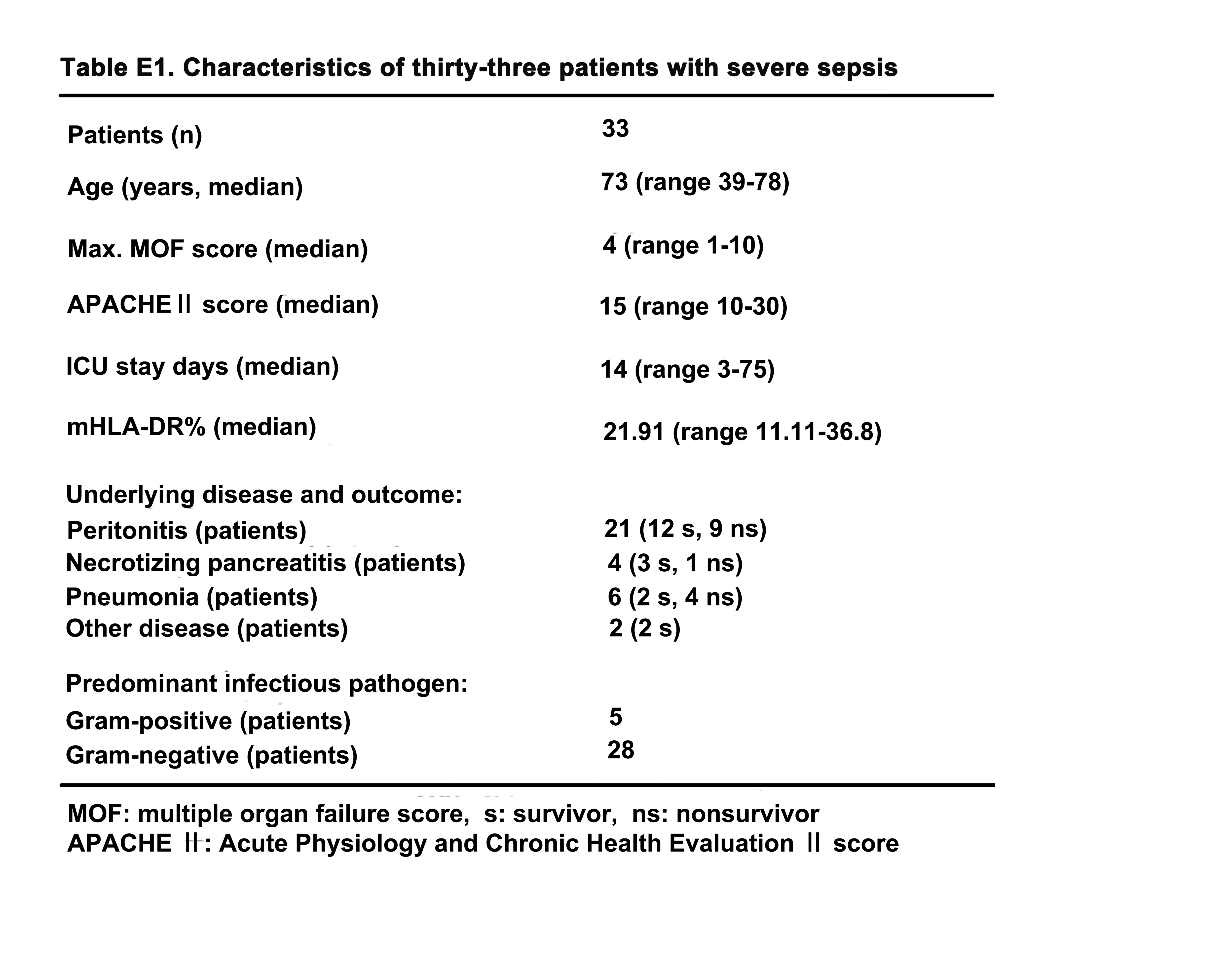
**

**Supplementary Table S2. PCR primers**

gene primers sequences (5' to 3')

human Forward CTAGTCTAGAGGAGGACGAACATCCAAC

T789 Reverse CGCGTCTAGAAAGCAAACTTTATTTCTCGC

human Forward CTAGTCTAGAGGAGGACGAACATCCAAC

T789m Mutated sense atttacaga*GTCCGTG*atttatttgggaga

ccgggg

Mutated antisense CCCAAATAAAT*cacggac*TCTGTAAATAAA TAAATAATAA ATAAATAAT

Reverse CGCGTCTAGAAAGCAAACTTTATTTCTCGC

human Forward CTAGTCTAGAGGAGGACGAACATCCAAC

T430 Reverse ATTATCTAGACTCCATGGGGAGGGCTGG

human Forward CATTTCTAGAgccagctccctctatttatg

T360 Reverse CGCGTCTAGAAAGCAAACTTTATTTCTCGC

human Forward CATTTCTAGAgccagctccctctatttatg

T142 Reverse ATTATCTAGAACATGTCTGAGCCAAGGCAG

human Forward CTAGGATTATTTATTATTTATTTATTATTTATT

T55 TATTTACAGATGAATGTATTTATTTG

Reverse CTAGCAAATAAATACATTCATCTGTAAATAA

ATAAATAATAAATAAATAATAAATAATC

human Forward CTAGGATTAT*G*TATTATTTATTTATTATTTATT

T55 TATTTACAGATGAATGTATTTATTTG

m1 Reverse CTAGCAAATAAATACATTCATCTGTAAATAA

ATAAATAATAAATAAATAATA*C*ATAATC

human Forward CTAGGATTATTTATTAT*G*TATTTATTATTTATT

T55 TATTTACAGATGAATGTATTTATTTG

m2 Reverse CTAGCAAATAAATACATTCATCTGTAAATAA

ATAAATAATAAATA*C*ATAATAAATAATC

human Forward CTAGGATTATTTATTATTTAT*G*TATTATTTATT

T55 TATTTACAGATGAATGTATTTATTTG

m3 Reverse CTAGCAAATAAATACATTCATCTGTAAATAA

ATAAATAATA*C*ATAAATAATAAATAATC

human Forward CTAGGATTATTTATTATTTATTTATTAT*G*TATT

T55 TATTTACAGATGAATGTATTTATTTG

m4 Reverse CTAGCAAATAAATACATTCATCTGTAAATAA

ATA*C*ATAATAAATAAATAATAAATAATC

human Forward CTAGGATTATTTATTATTTATTTATTATTTAT*G*

T55 TATTTACAGATGAATGTATTTATTTG

m5 Reverse CTAGCAAATAAATACATTCATCTGTAAATA*C*

ATAAATAATAAATAAATAATAAATAATC

human Forward CTAGGATTATTTATTATTTATTTATTATTTATT

T55 TAT*G*TACAGATGAATGTATTTATTTG

m6 Reverse CTAGCAAATAAATACATTCATCTGTA*C*ATAA

ATAAATAATAAATAAATAATAAATAATC

human Forward CTAGGATTATTTATTATTTATTTATTATTTATT

T55 TATTTACAGATGAATGTAT*G*TATTTG

m7 Reverse CTAGCAAATA*C*ATACATTCATCTGTAAATAA

ATAAATAATAAATAAATAATAAATAATC

human Forward CTAGGATTAT*G*TATTAT*G*TAT*G*TATTAT*G*TAT*G*

T55 TAT*G*TACAGATGAATGTAT*G*TATTTG

FM Reverse CTAGCAAATA*C*ATACATTCATCTGTA*C*ATA*C*

ATA*C*ATAATA*C*ATA*C*ATAATA*C*ATAATC

human Forward CTAGGATTATTTATTATTTATTTATTATTTATT

T55 TATTTACAGA*GUCCGUG*ATTTATTTG

181BSM Reverse CTAGCAAATAAAT*CACGGAC*TCTGTAAATAA

ATAAATAATAAATAAATAATAAATAATC

human Forward TTTCTCGAGAGCTCCTGGGAGATATGGCC

TNFP

-991/+1 Reverse TTTAAGCTTGGGTGTGCCAACAACTGCC

human Forward TTTCTCGAGAGCTCCTGGGAGATATGGCC

TNFPm Mutated sense agtcgagtat*gtgtaccacg*ccttaacga

-991/+1 agacagggcc

Mutated antisense TTCGTTAAGG*CGTGGTACAC*ATACTCGACT

TCCATAGCCC

Reverse TTTAAGCTTGGGTGTGCCAACAACTGCC

human Forward TTTCTCGAGAAAGAAGAAGGCCTGCCC

TNFP

-656/+1 Reverse TTTAAGCTTGGGTGTGCCAACAACTGCC

human Forward TTTCTCGAGCCTACACACAAATCAGTCAG

TNFP TGG

-284/+1 Reverse TTTAAGCTTGGGTGTGCCAACAACTGCC

human Forward TTTCTCGAGGGAAGTTTTCCGCTGGTTG

TNFP

-83/+1 Reverse TTTAAGCTTGGGTGTGCCAACAACTGCC

human Forward TAATGGTACCGGCGTCTATAGAGCAGGC

CDP1 Reverse CGGCAAGCTTGCAACGTGGAGATAAACAAG

human Forward TAATGGTACCGGCGTCTATAGAGCAGGC

CDP1m Mutated sense TC*ATCATAATC*GGGCTC*ATCATAATC*CGTC

C*ATCATAATC*CGGCCGGGTTCCGTTGCC

Mutated antisense CG*GATTATGAT*GGACG*GATTATGAT*GAGC

CC*GATTATGAT*GAGCCCCACCCACAGTCT

Reverse CGGCAAGCTTGCAACGTGGAGATAAACAAG

human Forward TAATGGTACCATCACGGCTCACTGCAGC

CDP2 Reverse CGGCAAGCTTGCAACGTGGAGATAAACAAG

human Forward CCGCTCGAGCGATGTCTAATGGTTATGAAG

HuR Reverse CCCAAGCTTTTATTTGTGGGACTTGTTGG

human Forward ATGTGAGCTCCATTTGCTGAGCATGTACTG

IL-1α

3’UTR Reverse GCGCTCTAGATAACATTATGGTCTGATCAC

human Forward ATATGAGCTCAGAGAGCTGTACCCAGAGAG

IL-1β

3’UTR Reverse CCGTTCTAGATAAAGAGAGCACACCAGTCC

human Forward Tattttaattatttttaatttattaatatttaa

IL-6 atatgtgaagctgagttaatttatgtaagtc

3’UTR atattta

Reverse CTAGTAAATATGACTTACATAAATTAACTCA

GCTTCACATATTTAAATATTAATAAATTAAAA

ATAATTAAAATAAGCT

Mouse Forward tgttgaatgttatttttaagttattttatctat

IL-1α gtatttataaatatatttatgataattatatta

3’UTR tttat

Reverse CTAGATAAATAATATAATTATCATAAATATATT

TATAAATACATAGATAAAATAACTTAAAAATAA

CATTCAACAAGCT

mouse Forward ctatttatatttgcacttattatttattattta

TNF-α tttattatttatttatttgcttatgaatgtatt

3’UTR tattt

Reverse CTAGAAATAAATACATTCATAAGCAAATAAA

TAAATAATAAATAAATAATAAATAATAAGTGC

AAATATAAATAGAGCT

**Supplementary Table S3. Sequences for small interference RNA**

gene sequences (5' to 3')

human sense GUUACUACCUCUUAUCCAUdTdT

Egr-1 antisense AUGGAUAAGAGGUAGUAACdTdT

human sense AAGCCUGUUCAGCAGCAUUGG

HuR antisense CCAAUGCUGCUGAACAGGCUU

mouse sense AAGAGGCAAUUACCAGUUUCA

HuR antisense UGAAACUGGUAAUUGCCUCUU

human sense CUGGGCUAACAGAACAACUAA

TIA-1 antisense UUAGUUGUUCUGUUAGCCCAG

human sense GAUUGAGGAGAAACGUAAAdTdT

P65 antisense UUUACGUUUCUCCUCAAUCdTdT

negative sense UUCUCCGAACGUGUCACGUdTdT

control

siRNA antisense ACGUGACACGUUCGGAGAAdTdT

**Supplementary Table S4. Primers for Q-PCR**

gene primers sequences (5' to 3')

human Forward CAGACTTCCTTGAGACACGG

TNF-α Reverse CAAGGCAGCTCCTACATTGG

mouse Forward CATCTTCTCAAAATTCGAGTGACAA

TNF-α Reverse TGGGAGTAGACAAGGTACAACCC

human Forward CACCATCTTCCAGGAGCGAG

GAPDH Reverse GCAGGAGGCATTGCTGAT

mouse Forward CTGCAAGAGACTTCCATCCAGTT

IL-6 Reverse GAAGTAGGGAAGGCCGTGG

mouse Forward TCAGCACCTTACACCTACCAG

IL-1α Reverse CAACTCCTTCAGCAACACGG

mouse Forward CGGCACACCCACCCTG

IL-1β Reverse AAACCGTTTTTCCATCTTCTTCT

mouse Forward TCAGCGTTCCAACAGCCTCA

IL-12α Reverse TGGTCTTCAGCAGGTTTCGG

Mouse Forward GGGACATCATCAAACCAGACC

IL-12β Reverse GCCAACCAAGCAGAAGACAGC

mouse Forward CAATGAACGCTACACACTGC

IFN-γ Reverse CCACATCTATGCCACTTGAG

human Forward GTGGAGACCATCAAGGAAGAC

IFN-γ Reverse TGCGTTGGACATTCAAGTC

human Forward CAGCCACTACAAGCAGCACT

GM-CSF Reverse TGACAAGCAGAAAGTCCTTCAG

human Forward GGGTGACACACTATGGTATTTGAG

IL-10 Reverse CTCCCTGGTTTCTCTTCCTAAG

mouse Forward ATGAACTTGGACCTCTGCGG

IL-2 Reverse GGGCTTGTTGAGATGATGCTT

human Forward GAGCACAGATACCACCAAGAC

P65 Reverse TGGGATGAGAAAGGACAGGC

mouse Forward AGGCACCAATGAGAACGGAC

HuR Reverse AGTATCTGATGTGTGGGCACC

human Forward attttcatctacaacctggg

HuR Reverse TTATTTGTGGGACTTGTTGG

mouse Forward AGAGGGAAATCGTGCGTGAC

β-actin Reverse AGGAGCCAGAGCAGTAATCTC

human Forward TTGTTACAGGAAGTCCCTTGCC

β-actin Reverse ATGCTATCACCTCCCCTGTGTG

human Forward ACCCTAAGCTGGAGGAGATG

Egr-1 Reverse GGTTGGTCATGCTCACTAGG

human Forward TTCCGCAAGTTCCACTATCTC

HLA-DRα Reverse TCCCAATAATGATGCCCAC

human Forward GCAAGATGCTGAGTGGAGTC

HLA-DRβ Reverse AATCCTGTTGGCTGAAGTCC

human Forward CTCTTCATGCTCTCTGGTTC

pri-miR-

181c/d Reverse AACAGGCTGAGTGACTGTG

RT CTCAACTGGTGTCGTGGAGTCGGCAATTCAGTT

GAGACTCACCG

miR-181a Forward ACACTCCAGCTGGGAACATTCAACGCTGTCG

Reverse TGGTGTCGTGGAGTCG

RT CTCAACTGGTGTCGTGGAGTCGGCAATTCAGTT

GAGACCCACCG

miR-181b Forward ACACTCCAGCTGGGAACATTCATTGCTGTCG

Reverse TGGTGTCGTGGAGTCG

RT CTCAACTGGTGTCGTGGAGTCGGCAATTCAGTT

GAGACTCACCG

miR-181c Forward ACACTCCAGCTGGGAACATTCAACCTGTCG

Reverse TGGTGTCGTGGAGTCG

RT CTCAACTGGTGTCGTGGAGTCGGCAATTCAGTT

GAGACCCACCG

miR-181d Forward ACACTCCAGCTGGGAACATTCATTGTTGTCG

Reverse TGGTGTCGTGGAGTCG

RT CTCAACTGGTGTCGTGGAGTCGGCAATTCAGTT

GAGCGCCAATA

miR-16 Forward ACACTCCAGCTGGGTAGCAGCACGTAAATA

Reverse TGGTGTCGTGGAGTCG

RT AACGCTTCACGAATTTGCGT

U6 Forward CTCGCTTCGGCAGCACA

Reverse AACGCTTCACGAATTTGCGT

**Supplementary References**

Esposito AM, Mateyak M, He D, Lewis M, Sasikumar AN, Hutton J, Copeland PR, Kinzy TG (2010) Eukaryotic Polyribosome Profile Analysis. *Journal of visualized experiments: JoVE*

Liao B, Hu Y, Brewer G (2007) Competitive binding of AUF1 and TIAR to MYC mRNA controls its translation. *Nature structural & molecular biology* **14:** 511-518
