# Supplementary material for: Modulation of TNF-α mRNA stability by human antigen R and miR181s in sepsis-induced immunoparalysis
Source: EMBO Mol Med. 2014 Dec 22;7(2):140–57. doi: 10.15252/emmm.201404797 (PMC4328645; doi:10.15252/emmm.201404797)
Supplement: Supplementary file 2 [file emmm0007-0140-sd2.pdf]

EMBO
Molecular Medicine

Modulation of TNF-α mRNA Stability by Human Antigen R and miR181s in Sepsis-Induced Immunoparalysis

Cao Dan, Bian Jinjun, Hua Zi-Chun, Ma Lin, Chen Wei, Zhang Xu,Zhou Ri, Cheng Shun, Sun Wen-Zhu, Jiao Qing-Cai, Yin Wu

Corresponding author: Wu Yin, State Key Laboratory of Pharmaceutical Biotechnology

Review timeline:

Submission date:	28 October 2014
Accepted:	03 December 2014

Editor: Céline Carret

Transaction Report:

No Peer Review Process File is available with this article, as the authors have chosen not to make the review process public in this case.
